# The Antarctic Wintering Alters the Properties of Human Plasma Cell-Free DNA

**DOI:** 10.1155/bri/8994730

**Published:** 2025-09-09

**Authors:** Sergey Ponomarev, Anastasia Kotikova, Nikolay Osetsky, Natalia Veiko, Elizaveta Ershova, Elena Malinovskaya, Ekaterina Savinova, Julia Chudakova, Julia Eliseeva, Vera Izhevskaia, Sergey Kutsev, Svetlana Kostyuk

**Affiliations:** ^1^Laboratory of Immune System Physiology, Russian Federation State Research Center Institute of Biomedical Problems RAS (IMBP RAS), Moscow, Russia; ^2^Chair of Biochemistry and Molecular Biology, Pirogov Russian National Research Medical University, Moscow, Russia; ^3^Laboratory of Molecular Biology, Research Centre for Medical Genetics (RCMG), Moscow, Russia; ^4^Institute of Longevity with a Clinic of Rehabilitation and Preventive Medicine, Petrovsky National Research Center of Surgery (NRCS), Moscow, Russia; ^5^Department of Biology and General Genetics, Institute of Medicine, Patrice Lumumba Peoples' Friendship University of Russia (RUDN), Moscow, Russia

**Keywords:** cell-free DNA, confinement, rDNA, SatIII, TLR9, wintering

## Abstract

Extracellular DNA of blood plasma (cell-free DNA, cfDNA) may potentially indicate a total level of apoptosis and mediate the immune response to stress induced by extreme environmental conditions, such as Antarctic wintering. We studied blood nuclease activity (NA); the content of 8-oxodG, rDNA, and SatIII(1q12) in cfDNA; and the levels of BAX, BCL2, TLR9, AIM2, STING, RIG-I, NF-kB, IL-8, and IL-17A mRNAs in 11 males, the members of the 64th Russian Antarctic Expedition. Blood was sampled before the wintering and on the 27th, 85th, 160th, 270th, and 315th days. The early months of the wintering are characterized by increased rates of apoptosis, an elevated BAX/BCL2 RNA ratio in blood leukocytes, and high cfDNA concentrations and NA in blood plasma. The properties of cfDNA are dramatically changed: the content of GC-rich rDNA rises, while AT-rich SatIII and 8-oxodG are low. We note individual multidirectional changes in the expression of *TLR9* and *AIM2*, while *STING* and *RIG-I* are downregulated in all of the subjects. The mRNA levels of *NFKB1*, *IL-8*, and *IL-17A* increase dramatically, indicating immune system activation. In conclusion, (1) apoptosis is overactivated and remains elevated during the first half of the Antarctic wintering; (2) cfDNA is enriched with GC-repeats, which stimulates its biological activity; and (3) the expression of immunity genes associated with the inflammatory response is increased.

## 1. Introduction

Antarctic wintering can be considered a model of certain space flight factors. The Antarctic environment includes isolation, cold, low atmospheric pressure, UV radiation, an altered geomagnetic field, and circadian rhythm disruption. These significantly influence the physiological systems of the human body, inducing acute and chronic stress [1]. Members of Antarctic expeditions also suffer from emotional stress caused by physical confinement, social deprivation, and fear of the unknown [2].

Stress evokes two basic responses in the organism: adaptation and programmed cell death. We attempt to estimate the two processes by measuring apoptotic gene expression, cell-free DNA (cfDNA) and its properties, and immune gene expression.

The main executors of programmed cell death are the proteins of the BCL-2 family, namely, BAX and BCL-2. BAX codes for Bcl-2 associated X protein being the main activator of p53-mediated apoptosis [3], and BCL2 expresses B-cell lymphoma 2 protein being the key inhibitor of apoptosis [4]. Thus, the BAX/BCL2 mRNA ratio reflects the level of cell death in a cell population [5].

The DNA of dead cells enters the circulatory system forming the pool of cfDNA, which may be extracted from the blood plasma to assess apoptosis rates. It is widely used in clinical practice for diagnosis confirmation, disease prognosis, and medical observation. The concentration of cfDNA increases during sepsis, cancer, cardiovascular conditions, autoimmune reactions, and mental illnesses [6–15]. Pregnancy, physical activity, and psychosocial stress are also known to elevate cfDNA [16–20].

The increase in cfDNA is followed by the activation of blood nucleases, which cut it into fragments to be excreted with urine [21–24]. Because blood nuclease activity (NA) balances the concentration of cfDNA provided by cell death, we measure DNase I activity in the blood for a more appropriate estimation of apoptosis rates [13, 21, 25].

Some authors consider cfDNA not only as a diagnostic biomarker of pathology but also as a potential therapeutic target, for it demonstrates pronounced biological activity determined by its molecular characteristics distinct from those of the intracellular genomic DNA (gDNA) [6, 26–39]. Firstly, cfDNA is more oxidized than gDNA [13, 32, 36], which increases its ability to penetrate cell membranes [34, 40]. The fragments of cfDNA in cytoplasm may potentially induce the signaling pathways of nucleic acid sensing receptors discussed below. Secondly, the GC content in cfDNA significantly differs from that of gDNA [41]. In healthy donors, cfDNA extracted from blood plasma contains approximately 54% GC, compared to 42% GC in gDNA [42]. During pathologies or conditions accompanied by chronic oxidative stress, cfDNA consists predominantly of GC-rich DNA fragments such as mitochondrial DNA and ribosomal DNA (rDNA) [30, 43–47].

Immunity activation may be triggered by cfDNA through the activation of so-called DNA sensors: TLR9, AIM2, STING, and RIG-I. High GC content in cfDNA facilitates its binding to TLR9, which then induces an intracellular signaling cascade activating the transcription factor NF-kB. The latter is known to regulate the immune response, apoptosis, and cell cycle [48, 49]. Another innate immunity protein, AIM2, is a sensor of AT-rich double-stranded DNA in cytoplasm. The aberrant activation of AIM2 by cytoplasmic DNA is thought to be a motive force of inflammation [50–52]. In addition to TLR9 and AIM2, cGAS is able to bind cytosolic DNA, including cfDNA fragments which penetrated the cell, and to activate a pathway leading to interferon synthesis. A key protein in this pathway, STING, takes part in the activation of, among other proteins, NF-kB. DNA sensing through cGAS/STING is believed to have a key role in inflammation [53–55]. The RIG-I protein encoded by the DDX58 gene recognizes exogenic RNA molecules in cell cytoplasm. It interacts with the 5′-triphosphate on the nascent double-stranded RNA transcribed from AT-rich DNA by RNA polymerase III (PolIII) [56, 57]. When cfDNA penetrates the cell and is transcribed into dsRNAs, which are recognized by RIG-I, the induced signaling pathway leads to the expression of type-I interferons. Of note, the mediators in this signaling cascade, IRF3 and IRF7, interact with NF-kB in the nucleus [58].

Since the transcription factor NF-kB plays a substantial role in the signal transduction cascades from cfDNA to the cell nucleus leading to the synthesis of a vast spectrum of cytokines, its overexpression during stress conditions may be considered a major sign of immunity activation. Proinflammatory cytokines, such as IL-8 and IL-17, evidence inflammation.

Considering cfDNA as a main coordinator between apoptosis and adaptation to Antarctic conditions, we analyze its concentration, properties (oxidation and GC/AT content), and the nuclease activity (NA) in blood plasma of the members of a 1-year expedition at Vostok Station. We also compare these indices with the expression levels of the genes regulating apoptosis (*BAX* and *BCL2*), the genes of DNA sensors (*TLR9*, *AIM2*, *STING*, and *RIG-I*), and genes associated with proinflammatory NF-kB activity (*NFKB1*, *IL8*, and *IL-17A*) in blood leukocytes.

## 2. Materials and Methods

### 2.1. Experiment Design

**Figure 1:**
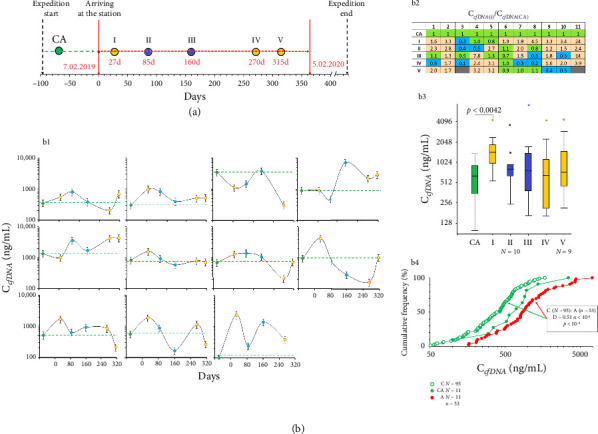
Plasma cfDNA concentrations. (a) The overall plan of the 64th Russian Antarctic Expedition and the time intervals between the blood sampling time points. (b) The changes in the concentrations of cfDNA (C_cfDNA_) in the blood plasma of the expedition members during their stay at Vostok Station; b1—cfDNA concentrations in the blood samples of the subjects (1–11) at the indicated time points (mean values and SE); green columns indicate samples obtained before the arrival at the station (CA), yellow columns indicate samples obtained during polar day, and blue columns—samples obtained during polar night; b2—the analysis of the changes in blood C_cfDNA_ of the subjects during their stay at Vostok Station (i) compared to control (CA); green cells—C_cfDNA_ did not change (*p* > 0.05, *U*-test), brown cells—C_cfDNA_ increased (*p* < 0.05), and blue cells—C_cfDNA_ decreased (*p* < 0.05); b3—the analysis of the changes in blood C_cfDNA_ of 10 subjects at the indicated time points; horizontal lines indicated medians (*U*-test); b4—the distribution of plasma C_cfDNA_ samples from healthy male donors not subjected to extreme stress (control, C) and from the group under study before (CA) and during the wintering period (A); in the frame: the comparison of the distributions of the groups C and CA using the Kolmogorov–Smirnov test (D, *α*) and the Mann–Whitney *U* test (p).

The study was carried out during the 64th Russian Antarctic Expedition, which lasted from November 7, 2018, to June 6, 2020. Within this period, the participants of the expedition inhabited Vostok Station from February 7, 2019, to February 5, 2020 (Figure 1(a)). The study included male participants of the wintering (*n* = 11) aged from 32 to 64 (mean 49.7 ± 10.4 years). All participants were admitted by the medical expert commission and signed informed consent for participation in the study. The study was approved by the Bioethics Committee of SSC RF-IBMP RAS (protocol No 487 from October 11, 2018). The mean height and body mass were 174.3 ± 1.9 cm and 80.8 ± 2.9 kg, respectively. The physical activity of the expedition members during their stay at the station remained low during the whole wintering period, excluding the recurrent (1-2 times a week) snow stockpiling for the maintenance of the water supply of the station. The state of the participants remained satisfactory during the whole wintering period.

The expedition doctor sampled blood from all the participants before their arrival at the station (samples CA, control) and at several time points during the wintering period (I-V). Samples I (6 Mar 2019), IV (5 Nov 2019), and V (18 Dec 2019) allow us to estimate the parameters during the conditions of the polar day, while samples II (3 May 2019) and III (16 July 2019) reflect the response to the polar night conditions.

Immediately after blood sampling, the plasma was fractionated from cells by centrifugation, and the erythrocytes were lysed and separated from white blood cells. The samples of both plasma and leukocyte mass were frozen. In total, 64 samples of blood plasma and 64 samples of leukocyte mass were analyzed upon delivery to Moscow. Two samples (time point V, participants #3 and #11) were not obtained for technical reasons.

The control group consisted of 95 healthy males (age 41 ± 15) with no history of any disorder or stress a month before blood sampling.

### 2.2. cfDNA Extraction and Measurement of Concentration

Phenol extraction with the prior hydrolysis of plasma and subsequent RNase A and protease K treatment was shown to be an optimal method for the analysis of cfDNA concentration [23]. 0.1 V of lysis buffer (10% sodium lauryl sarcosylate, 0.075 mg/mL RNase A [Sigma, USA], 0.2 M EDTA) was added to plasma, incubated for 1 h at 37°C, then treated with protease K 0.2 mg/mL (Promega, USA) for 24 h at 37°C. After two purification cycles using a saturated phenol solution, cfDNA fragments were precipitated in ethanol and 2 M ammonium acetate. The precipitate was then washed twice with 75% ethanol, dried, and dissolved in water. cfDNA concentration was determined after staining the samples with PicoGreen dye (Molecular Probes/Invitrogen, CA, USA) by measuring the fluorescence on EnSpire Plate Reader (PerkinElmer, Waltham, MA, USA) at excitation and emission wavelengths of 488 and 528 nm, respectively. The cfDNA concentration in the sample was calculated according to a DNA standard curve. The standard error for the assessment of cfDNA concentration in water solution by fluorescence was 3%–5%. The total error, including the step of DNA isolation, was 9 ± 5%.

### 2.3. Nuclease Activity Assessment

To assess NA, we applied a method of radial diffusion in agarose gel stained with EtBr [59]. To calculate NA, a calibration dependence was obtained, which relates the fluorescence of the dye in the spot with the concentration of the standard DNase I sample (Sigma, USA) in solution. The result is given in units of activity (U/mL). 1 unit corresponds to the activity of DNase I, taken at a concentration of 1 ng/mL (1 h, 37°C). At least three parallel measurements were made for one sample. The relative standard error of the method was 5%.

### 2.4. The Estimation of cfDNA Oxidation

The oxidation level of cfDNA was determined by ELISA with the anti-8-oxodG antibodies [11, 13, 35]. Briefly, the DNA samples were applied to a filter (Optitran BA-S85, GE Healthcare), with three dots (10 ng/dot) per sample. Four standard samples of oxidized gDNA (10 ng/dot) with the known concentration of 8-oxodG (determined by ESI-MS/MS on AB SCIEX 3200 Qtrap) were applied to the same filter to obtain a calibration dependence relating the signal intensity to the copy number in a sample. The filters were heated at 80°C in a vacuum for 1.5 h. For detection, alkaline phosphatase-conjugated anti-8-oxodG antibody (Abcam) and the corresponding substrates NBT and BCIP were used. For quantification analysis, Images 6.0 software (RCMG, Russia) was used. The relative standard error of the method was 15 ± 5%.

### 2.5. Nonradioactive Quantitative Hybridization (NQH)

The method of quantitative nonradioactive hybridization was specified in detail previously [60]. Briefly, the denatured cfDNA samples (50 ng/mL) were applied to a prepared filter (Optitran BA-S85, GE Healthcare) along with the standard samples of the gDNA (50 ng/mL) with a known content of the rDNA or SatIII to plot a calibration curve for the dependence of the signal intensity on the number of rDNA copies (or SatIII content) in a particular sample. Lambda phage DNA (50 ng/mL) was also applied to the same filter to control the nonspecific signal. The filter was heated at 80°C in a vacuum for 1.5 h, then hybridized with the corresponding probes, dried, scanned, and analyzed using Images 6.0 software (RCMG, Russia). The software determined the dot location, measured the nearest background signal, and calculated the integral dot intensity. Signals from several dots corresponding to the same sample were averaged. The rDNA or SatIII content in a studied DNA sample was calculated using the calibration curve equation. The relative standard error was 11 ± 8%.

**Figure 2:**
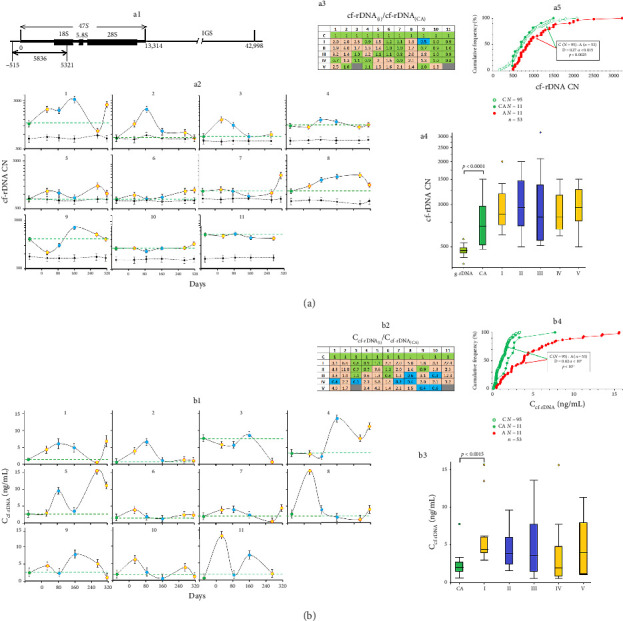
The content of GC-rich fragments in the plasma of the volunteers, subjected to the Antarctic conditions. (a) The content of rDNA in the plasma samples of the subjects; a1—the scheme of the rDNA repeat with an arrow indicating a probe, and the changes in the cell-free rDNA; a2—the content of rDNA in the plasma samples of the subjects (1–11) at the indicated time points (mean values of four measurements and SE) compared to gDNA; a3—the analysis of the changes in the content of cell-free rDNA of the subjects during their stay at Vostok Station (i) compared to control (CA); a4—the analysis of the changes in plasma rDNA of 11 subjects at the indicated time points; horizontal lines indicate medians; a5—the distribution of rDNA content in the samples from healthy male donors not subjected to extreme stress (control, C) and from the group under study before (CA) and during the wintering period (A); in the frame: the comparison of the distributions of the groups C and CA using the Kolmogorov–Smirnov test; (b) the changes in the cf-rDNA concentrations (C_cf-rDNA_) in the subjects during the wintering; b1—the concentration of rDNA in the plasma samples of the subjects (1–11) at the indicated time points; b2—the analysis of the changes in the concentration of cell-free rDNA of the subjects during their stay at Vostok Station (i) compared to control (CA); b3—the analysis of the changes in C_cf-rDNA_ of 11 subjects at the indicated time points; horizontal lines indicate medians; b4—the distribution of rDNA concentration in the samples from healthy male donors not subjected to extreme stress (control, C) and from the group under study before (CA) and during the wintering period (A); in the frame: the comparison of the distributions of the groups C and CA using the Kolmogorov–Smirnov test.

For the detection of the human ribosomal repeat, the probe p (ETS-18S) (the fragment of rDNA 5.8 kb long, from −515 to 5321, relative to the transcription initiation point, HSU 13369, GeneBank) was used (Figure 2(a)). The f-SatIII probe was a 1.77 kb cloned EcoRI fragment of human satellite III DNA. Dr. H. Cook (MRC, Edinburgh, UK) kindly provided the human chromosome 1q12-specific repetitive satellite DNA probe pUC1.77. The DNA probes were biotinylated using the Biotin NT Labeling Kit (Jena Bioscience GmbH).

### 2.6. RNA Extraction, Reverse Transcription, and PCR

RNA was extracted from cells using YellowSolve kits (Klonogen, St. Petersburg, Russia) or Trizol reagent (Invitrogen, Carlsbad, CA, USA) according to the manufacturers' instructions. The RNA concentration was determined using a QuantiT RiboGreen RNA reagent dye (R11491, Invitrogen, Carlsbad, CA, USA) on EnSpire plate reader (PerkinElmer, Waltham, MA, USA). According to the standard protocol, the reverse transcription reaction was performed using reagents from Sileks (Moscow, Russia). PCR was performed using the specific primers, SYBR Green intercalating dye (Molecular Probes/Invitrogen, CA, USA), and StepOnePlus device and software (Applied Biosystems, Foster City, CA, USA). Evrogen (Moscow, Russia) performed the primer design and synthesis. The following primers were used (see Table 1).

The PCR mixture for one reaction consisted of 1Х PCR buffer (70 mmol Tris-HCl, pH 8.6; 16 mmol ammonium sulfate, 3.5 mmol MgCl_2_), 0.1 mmol dNTP, 1 pmol of primers, and cDNA. The run consisted of denaturation for 4 min at 95°C, 40 amplification cycles: 94°C for 20 s, 56°C–62°C for 30 s, 72°C for 30 s, and final elongation at 72°C for 5 min. The results were processed using a calibration plot with CA as a reference sample and TBP as a reference gene. The error was 2%.

### 2.7. Statistical Analysis

All data within the manuscript are presented as mean ± SE. Data were analyzed using the Mann–Whitney *U* test and/or Bonferroni's multiple comparison test. *p* < 0.05 was considered statistically significant. The distributions of the samples by the parameter values were compared by the Kolmogorov–Smirnov method (D and *α*). The analysis of correlations between the parameters was performed by the Spearman rank correlation method (correlation coefficient Rs and probability p). The data were analyzed with Excel, Microsoft Office (Microsoft, Redmond, WA, USA), StatPlus 2007 (AnalystSoft, USA), and StatGraphics (Statgraphics Technologies, The Plains, VA, USA).

## 3. Results

### 3.1. Alterations in the Plasma cfDNA Concentration

Figure 1(b) demonstrates the concentrations of cfDNA (C_cfDNA_) of the participants during the wintering on Vostok Antarctic station at the indicated time points (panel b1) and the statistical analysis of their changes compared to the control (panel b2). In most cases, the blood concentration of cfDNA increased in 10 expedition members compared to the control (CA). Participant #3 showed an abnormally high plasma cfDNA concentration in the control sample, which then slightly decreased but was still relatively high. Apparently, it reflected a latent chronic kidney disease, which then manifested in acute form and caused the untimely evacuation of this participant from the station. Panel b3 in Figure 1(b) indicates a significant increase in C_cfDNA_ in all members at time point I, i.e., a month after the arrival at the station (the data for participant #3 were excluded from the analysis).

Panel b4 in Figure 1(b) demonstrates the comparison between the cfDNA concentrations of the Antarctic expedition members (Group A, 53 samples, *N* = 11) and of healthy males of approximately the same age who were not subjected to extreme environmental conditions (Group C, *N* = 95). Group A had elevated concentrations of cfDNA fragments in their blood.

Thus, the extreme Antarctic conditions cause a temporary elevation in the blood cfDNA concentration, especially during the first month of the wintering.

### 3.2. Alterations in the Plasma Nuclease Activity

**Figure 3:**
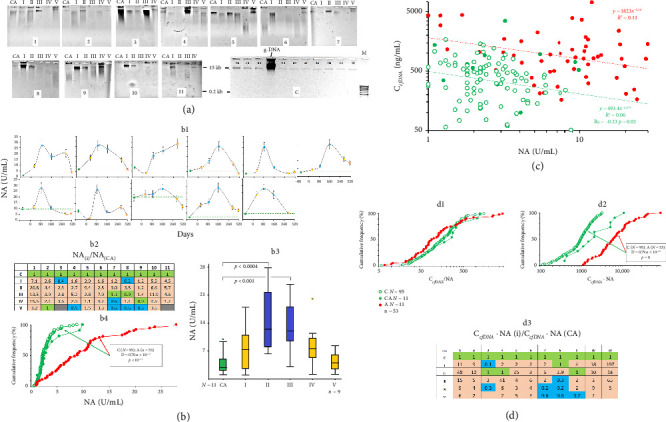
The analysis of the blood nuclease activity (NA) dynamics of the subjects during their 1-year stay in Antarctica. (a) The electrophoretograms of plasma cfDNA from the subjects (1–11) at the indicated time points and the control samples (C); (b) the analysis of plasma NA; b1—NA indices in the plasma samples of the subjects (1–11) at the indicated time points (mean values of four measurements and SE); b2—the analysis of the changes in blood NA of the subjects during their stay at Vostok Station (i) compared to control (CA); b3—the analysis of the changes in plasma NA of 11 subjects at the indicated time points; horizontal lines indicate medians (*U*-test); b4—the distribution of plasma NA samples from healthy male donors (C, green) and from the group under study during the wintering period (A, red); in the frame: the comparison of the distributions of the groups C and CA using the Kolmogorov–Smirnov test (D, *α*); (c) the dependency of C_cfDNA_ and NA toward each other in the three groups; (d) the distribution of plasma C_cfDNA_/NA samples (d1) and C_cfDNA_ ^∗^NA samples (d2) in three groups; d3—the analysis of the changes in C_cfDNA_ ^∗^NA of the subjects during their stay at Vostok Station (i) compared to control (CA).

Electrophoretograms in Figure 3(a) illustrate the sizes of the cfDNA fragments extracted from the blood plasma of the expedition members (Group A, #1–#11) and several control samples obtained from healthy donors (Group C). In the control samples, cfDNA consists of a low quantity of long (> 15 kb) DNA fragments, while in Group A, it contains 0.1–15 kb molecules presented in high amounts. This indicates a significant upregulation of blood nuclease activity in the expedition members, which may happen due to increased apoptosis rates, followed by an elevated amount of DNA entering the bloodstream.

Indeed, the plasma nuclease activity (NA) in Group A was especially pronounced during the first months after the arrival at the station, and it reached its maximal values at polar night (Figure 3(b), panels b1 and b2). During the second half of the wintering, plasma NA decreased in most participants (Figure 3(b), panel b3). In sum, people residing at Vostok Station demonstrated significantly higher blood plasma NA during their stay compared to that of healthy people in the absence of extreme stress conditions (Figure 3(b), panel b4).

For the control group, we found a negative correlation between blood NA and the concentration of cfDNA (Figure 3(c), Table 2): the higher the plasma nuclease activity was, the fewer cfDNA fragments resided in the blood. For Group A, this negative correlation was less pronounced. The high NA in the expedition members (> 10 U/mL, equal to the upper limit for the control group) was associated with the higher C_cfDNA_.

Recently, we proposed applying the C_cfDNA_/NA ratio to characterize cfDNA in people subjected to chronic stress, such as increased radiation [25]. This index was significantly lower for people exposed to radiation. Neither Group C nor Group A demonstrated significant changes in the C_cfDNA_/NA ratio (Figure 3(d), panel d1). However, we noted pronounced differences between the two groups when multiplying the indices C_cfDNA_ ∗ NA (Figure 3(d), panel d2). We conclude that Antarctic wintering causes an upregulation of nuclease activity in the blood with a simultaneous increase in cfDNA fragment concentration.

### 3.3. cfDNA Oxidation

**Figure 4:**
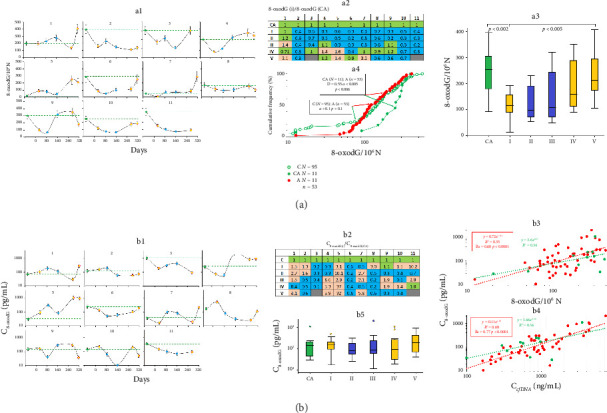
Oxidation of cfDNA during the Antarctic wintering. (a) The changes of 8-oxodG in plasma cfDNA; a1—the absolute content of 8-oxodG in the plasma samples of the subjects (1–11) at the indicated time points (mean values of three measurements and SE); a2—the analysis of the changes in the absolute values of 8-oxodG of the subjects during their stay at Vostok Station (i) compared to control (CA); a3—the analysis of the changes in 8-oxodG in plasma cfDNA of 11 subjects at the indicated time points; horizontal lines indicate medians; a4—the distribution of 8-oxodG content in the samples from healthy male donors not subjected to extreme stress (control, C) and from the group under study before (CA) and during the wintering period (A); in the frame: the comparison of the distributions of the groups C and CA using the Kolmogorov–Smirnov test; (b) the changes in 8-oxodG concentrations (C_8-oxodG_) in the blood of the subjects; b1—C_8-oxodG_ in the plasma of the subjects (1–11) at the indicated time points (mean values for three measurements and SE); b2—the analysis of the changes in C_8-oxodG_ of the subjects during their stay at Vostok Station (i) compared to control (CA); b3, b4—the dependency of 8-oxodG concentration from the absolute 8-oxodG content and the concentration of cfDNA (C_cfDNA_); b5—the analysis of the changes in plasma 8-oxodG concentration of 11 subjects at the indicated time points; horizontal lines indicate medians.

Ten of the subjects demonstrated a decreased level of the oxidation biomarker in their cfDNA during their stay on Vostok Station, while one of them, namely, expedition member #1, did not (Figure 4(a), panels a1–a3). We noted no significant differences in cfDNA oxidation between the control group and the group under study (Figure 4(a), panel a4). At the same time, the concentration of 8-oxodG in the blood varied individually (Figure 4(b), panels b1, b2, and b5). Thus, the biological action may develop depending on the level of 8-oxodG in cfDNA (Figure 4(b), panel b3) and the total concentration of cfDNA fragments in the blood (Figure 4(b), panel b4).

### 3.4. The Content of cf-rDNA in the Blood

GC content of cfDNA may significantly alter due to the accumulation of GC-rich fragments, such as rDNA repeat [41–45]. To analyze the level of extracellular rDNA in the cfDNA (cf-rDNA), we utilized the method of NQH [44, 46] and the DNA probe for the 73% GC-rich rDNA region (Figure 2(a)). Figure 2(a), panel a2, shows the rDNA copy number per genome equivalent of cfDNA; panel a3 demonstrates the analysis of rDNA content in the gDNA extracted from the same blood samples.

Figure 2(a), panel a4, demonstrates that cfDNA is more abundant in rDNA than gDNA, which is concordant with the published data [43, 44, 46]. In the subjects, the extracellular GC-rich rDNA fragments varied from 480 copies (in #5, CA) to 3200 copies (in #3, I) per diploid genome equivalent of cfDNA (mean 1015 ± 525; median 850; Kv = 0.52; *n* = 64). At the same time, gDNA contained from 380 (in #4) to 567 (in #2) rDNA copies per cell (mean 473 ± 32; median 472; Kv = 0.07; *n* = 64). Most of the expedition members had elevated rDNA levels at different time points of their mission, though subjects #10 and #11 had almost no changes in the cf-rDNA content (Figure 2(a), panel a3). In the group under study, we noted higher cf-rDNA levels compared to the control group (Figure 2(a), panel a5). The concentration of cf-rDNA (C_cf-rDNA_) increased in all of the subjects at different time points, except for subject #3, who initially demonstrated abnormally high C_cf-rDNA_ (Figure 2(b), panels b1–b4).

### 3.5. The Content of cf-SatIII in the Blood Plasma

**Figure 5:**
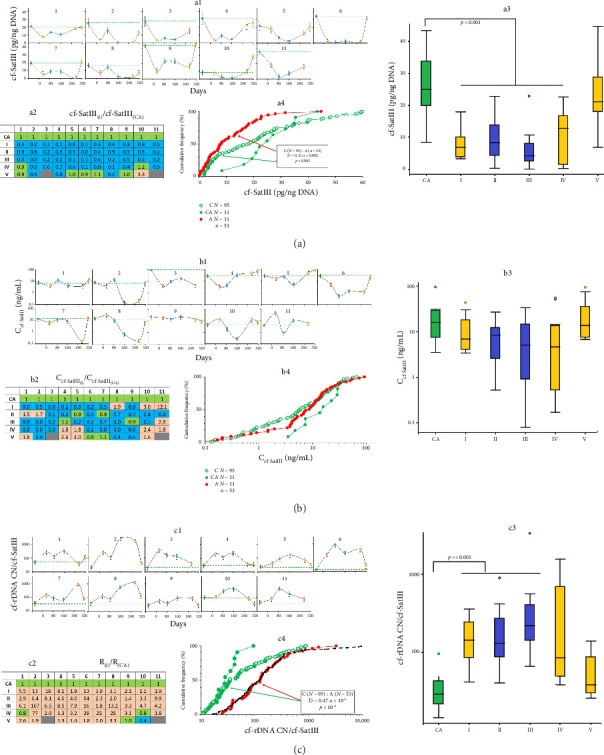
The content of AT-rich fragments in the plasma of the volunteers, subjected to the Antarctic conditions. (a) The changes in the cell-free SatIII; a1—the content of SatIII in the plasma samples of the subjects (1–11) at the indicated time points (mean values of four measurements and SE); a2—the analysis of the changes in the content of cf-SatIII of the subjects during their stay at Vostok Station (i) compared to control (CA); a3—the analysis of the changes in plasma SatIII of 11 subjects at the indicated time points; horizontal lines indicate medians; a4—the distribution of SatIII content in the samples from healthy male donors not subjected to extreme stress (control, C) and from the group under study before (CA) and during the wintering period (A); in the frame: the comparison of the distributions of the groups C and CA using the Kolmogorov–Smirnov test; (b) the changes in the cf-SatIII concentrations (C_cf-SatIII_) in the subjects during the wintering; b1—the concentration of cfSatIII in the plasma samples of the subjects (1–11) at the indicated time points; b2—the analysis of the changes in the concentration of cell-free SatIII of the subjects during their stay at Vostok Station (i) compared to control (CA); b3—the analysis of the changes in C_cf-SaIII_ of 11 subjects at the indicated time points; horizontal lines indicate medians; b4—the distribution of cf-SatIII concentration in the samples from healthy male donors not subjected to extreme stress (control, C) and from the group under study before (CA) and during the wintering period (A); in the frame: the comparison of the distributions of the groups C and CA using the Kolmogorov–Smirnov test; (c) the changes in the cell-free rDNA/SatIII ratio (*R* = cf-rDNA/cf-SatIII) in the subjects during the wintering; c1—the R value in the subjects (1–11) at the indicated time points; c2—the analysis of the changes in R during the wintering (i) compared to control (CA); c3—the analysis of the changes in R of 11 subjects at the indicated time points; horizontal lines indicate medians; c4—the distribution of R in healthy male donors not subjected to extreme stress (control, C) and the group under study before (CA) and during the wintering period (A); in the frame: the comparison of the distributions of the groups C and CA using the Kolmogorov–Smirnov test; the black dotted line indicates the distribution of R in the sample of people subjected to radiation [42].

We applied NQH for the analysis of Satellite III (1q12) content in cfDNA (cf-SatIII, Figure 5(a)), which contains 62% AT pairs. In the control group, cf-SatIII varied within a narrower range and was generally higher (mean 30 ± 10 pg/ng DNA; median 25 pg/ng DNA; Кv = 0.38; *n* = 11). In Group A, the content of cell-free SatIII and, thus, the AT percentage of cfDNA were lower. However, these indices normalized in half of the subjects by the end of the expedition.

The concentration of SatIII fragments in the blood plasma of the subjects (Ccf-SatIII) varied, and all of them demonstrated decreased Ccf-SatIII at different time points (Figure 5(b)). At the same time, this parameter did not differ between the control group and the group under study (Figure 5(b), panel b4).

In sum, the fraction of the extracellular DNA containing AT-rich fragments of SatIII was reduced in the blood of the expedition members with the simultaneous increase in GC-rich cf-rDNA. Earlier, we proposed the ratio of the two parameters (*R* = cf-rDNA/cf-SatIII) to assess the stress level caused by ionizing radiation [44]. The expedition members demonstrate elevated R during the whole wintering and, in general, a higher R compared to the control group (Figure 5(c)). Interestingly, the cumulative frequency for Group A samples coincides with that of the people subjected to radiation (black curve in Figure 5(c), panel c4) [44].

### 3.6. The Correlation Between the Characteristics of cfDNA

**Figure 6:**
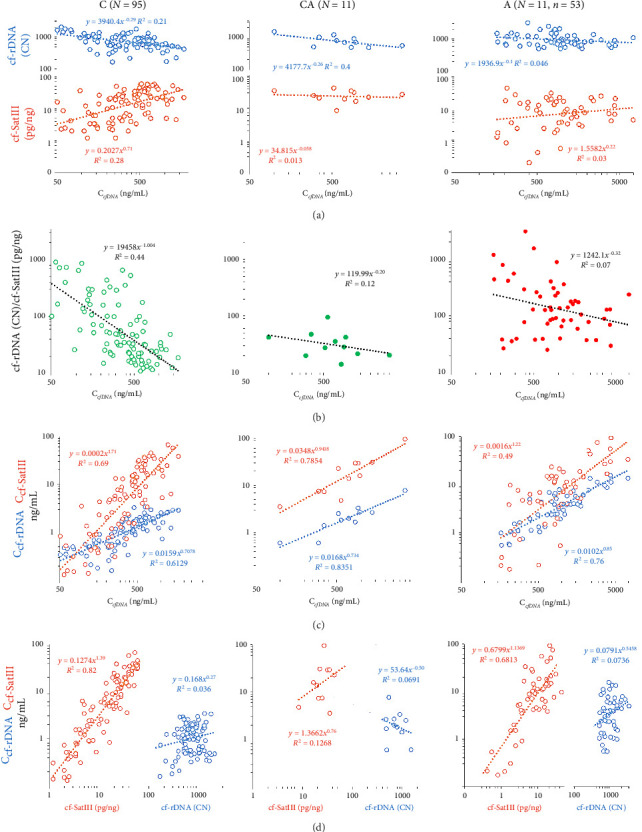
The correlations between the characteristics of plasma cfDNA. (a) The correlation between the circulating rDNA fragments (cf-rDNA), circulating SatIII fragments (cf-SatIII), and the total cfDNA concentration in the blood samples of the three groups; (b) the correlation between R (cf-rDNA/cf-SatIII) and the total cfDNA concentration in the blood samples of the three groups; (c) the correlation between the concentration of cf-rDNA, the concentration of cf-SatIII, and the total cfDNA concentration in the blood samples of the three groups; (d) the correlation between the circulating rDNA fragments (cf-rDNA), circulating SatIII fragments (cf-SatIII), and their content in the blood samples of the three groups.

Table 1 illustrates the results of the correlation analysis of the cfDNA parameters under study. Figures 6(a) and 6(b) demonstrate the dependence of cf-rDNA, cf-SatIII, and the R ratio on the total concentration of cfDNA in the blood. The control group shows a negative correlation between cf-rDNA, R, and С_cfDNA_ and a positive correlation between cf-SatIII and С_cfDNA_. The lower the cfDNA concentration is, the less disproportional GC- and AT-rich (or rDNA and SatIII) fragment concentrations appear to be. Similar trends were in the CA and A groups, though less pronounced (the correlations were nonsignificant, *p* > 0.05).

The concentrations of cf-rDNA and cf-SatIII depend on the total concentrations of the DNA fragments circulating in the blood plasma (Table 1, Figure 6(c)) and correlate positively. The concentration of cf-SatIII to a greater extent is defined by the absolute content of cf-SatIII. The concentration of cf-rDNA negatively correlates with the R ratio in both groups C and A.

### 3.7. The Expression of Genes Associated With Apoptosis

**Figure 7:**
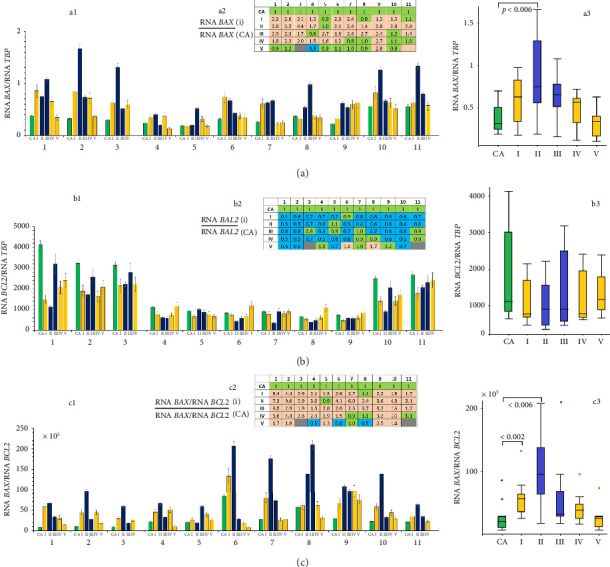
The analysis of pro- and antiapoptotic gene expression in the blood cells of the expedition members. (a) The analysis of *BAX* expression; a1—*BAX* mRNA levels in the leukocytes of the subjects (1–11) at the indicated time points; a2—the analysis of the changes in *BAX* expression compared to control; a3—the analysis of the changes in *BAX* expression in 11 subjects at the indicated time points; horizontal lines indicate medians; (b) the analysis of *BCL2* expression; b1—*BCL2* mRNA levels in the leukocytes of the subjects (1–11) at the indicated time points; b2—the analysis of the changes in *BCL2* expression compared to control; b3—the analysis of the changes in *BCL2* expression in 11 subjects at the indicated time points; horizontal lines indicate medians; (c) the assessment of *BAX*/*BCL2* mRNA ratio; c1—*BAX*/*BCL2* mRNA ratio of the subjects (1–11) at the indicated time points; c2—the analysis of the changes in *BAX*/*BCL2* ratio compared to control; c3—the analysis of the changes in *BAX*/*BCL2* mRNA ratio in 11 subjects at the indicated time points; horizontal lines indicate medians.

One of the reasons for the increased plasma cfDNA concentration and the alterations in GC/AT content, along with the upregulated nuclease activity, may be the high rate of cell deaths as a response to stress caused by the work in Antarctic conditions. To test this hypothesis, we analyzed the expression of the major pro- and antiapoptotic genes (*BAX* and *BCL2*, respectively) in the blood leukocytes of the expedition members using RT-qPCR (Figure 7). The mRNA level of *BAX* increased significantly in all of the expedition members and reached its maximum at time point II (the 85^th^ day of the wintering, Figure 7(a), panel a3). The expression of *BCL2* in leukocytes decreased in the subjects at different time points (Figure 7(b)). The *BAX*/*BCL2* RNA ratio characterizing the apoptosis level [5] was significantly increased at time points I and II (Figure 7(c)) and decreased by the end of the mission (Figure 7(c), panel c3).

**Figure 8:**
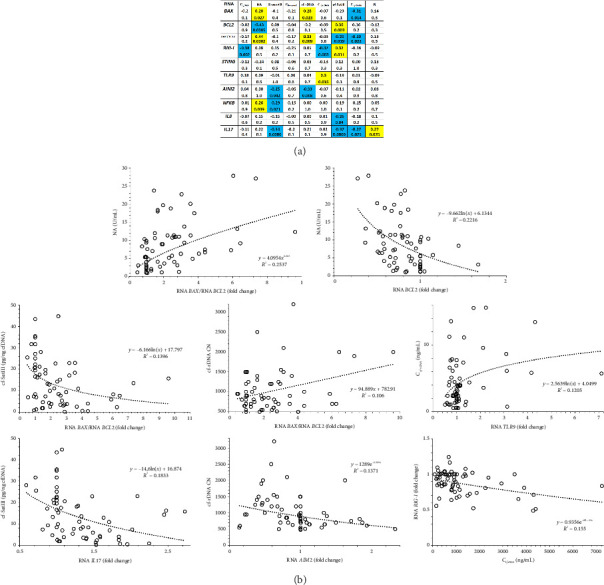
The correlations between the expression of the genes under study and the characteristics of plasma cfDNA. (a) The correlation between the cfDNA parameters and the mRNA levels of the genes in Group A (*n* = 64). (b) The correlations between the parameters characterized by the maximal correlation coefficients.

The alterations in *BAX* RNA levels positively correlated with NA and cf-rDNA and negatively correlated with cf-SatIII (Figure 8(a)). We noted negative correlations between *BCL2* RNA content in blood leukocytes and NA and a positive correlation between that and cf-SatIII. The higher the BAX/BCL2 RNA ratio was, the higher the NA and cf-rDNA parameters were. At this point, the content and concentration of the AT-rich fragment cf-SatIII remained low.  Table S1 reports the correlation between the parameters of cfDNA under study and the expression of the two genes.

### 3.8. The Gene Expression of DNA-Sensors

The alterations in the properties of the extracellular DNA are associated with its biological activity, realized through receptors capable of forming complexes with the cfDNA and/or mediating its influence on cell genome. We analyzed the mRNA expression of the four genes in charge of signal transmission from cfDNA to the nucleus, namely, *TLR9*, *AIM2*, *STING*, and *RIG-I* (Table 3).

5 out of 11 subjects, including subject #3, who showed abnormality before, had increased *TLR9* expression. In others, mRNA levels of *TLR9* either did not change or decreased 15%–50% at different time points of the wintering. These alterations positively correlated with the plasma concentration of the GC-rich ribosomal repeat (Figure 8) and the changes in the expression of the proapoptotic *BAX* gene (Table S1).

The mRNA levels of *AIM2* decreased in 6 volunteers at different time points up to 9 times (Table 3). They negatively correlated with the rDNA content in cfDNA (Figure 8).

*RIG-I (DDX58)* expression was 10%–50% lower in 10 expedition members compared to control values, while subject #11 did not demonstrate any significant changes. Of note, we did not note upregulation of this gene at any of the time points. These changes in the *RIG-I* mRNA level negatively correlated with the rDNA concentration and the total concentration of cfDNA and positively correlated with the concentration of cf-SatIII (Figure 8). Also, the expression of this gene negatively correlated with the alterations in DNA oxidation and TLR mRNA levels.

**Figure 9:**
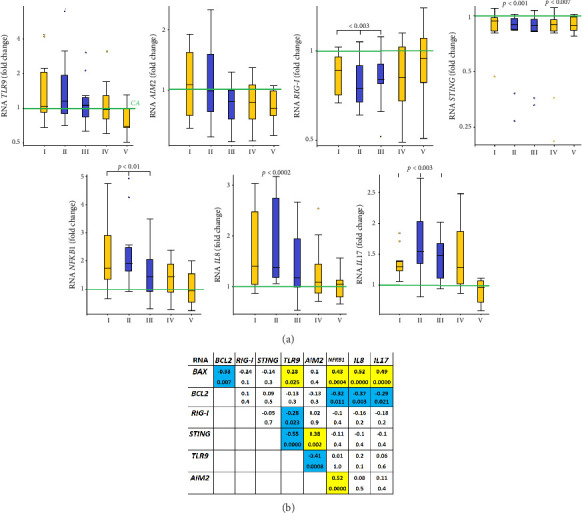
The analysis of the changes in the expression of DNA-sensor genes (*TLR9, AIM2, STING,* and *RIG-I*) and NF-kB associated genes (*NFKB1, IL8,* and *IL17A*). (a) The relative expression of the genes in the leukocytes from 11 subjects at the indicated time points; (b) the correlation analysis of mRNA levels of the indicated genes for the samples from Group A (*n* = 64).

*STING (TMEM173)* was either downregulated or unchanged during the wintering, though there was a significant negative correlation with *TLR9* expression (Figure 9(b)) and a positive correlation with *AIM2* mRNA levels.

### 3.9. The Alterations in the mRNA Levels of NFKB, IL8, and IL17A

The mRNA levels of the transcription factor *NF-kB*, as well as those of the *NF-kB*-controlled genes (*IL8* and *IL17A*), were elevated in the blood leukocytes of the expedition members during different time points of the Antarctic wintering (Table 3, Figures 8 and 9). These alterations in the expression of the three genes positively correlated with the changes in the proapoptotic *BAX* expression and negatively correlated with the expression of its antagonist *BCL2*. We also noted a pronounced positive correlation between the mRNA levels of *NFKB* and *AIM2*. In addition, the alterations in the expression of *NF-kB* positively correlated with NA (Figure 8(a)) and negatively correlated with cfDNA oxidation. There was a negative correlation between *IL8* mRNA levels and cf-SatIII and those of *IL17A* and cf-SatIII. The changes in the expression of *IL17A* also negatively correlated with the levels of 8-oxodG in cfDNA.

## 4. Discussion

### 4.1. Antarctic Conditions Increase Apoptosis

The first 2 months upon arrival at Concordia Station were shown to increase the level of ROS with the simultaneous downregulation of antioxidant systems [61], i.e., the Antarctic conditions stimulate oxidative stress, which, in turn, significantly impairs the functioning of immunity. It manifests as proinflammatory cytokines and the activation of T-cells, possibly mediated by latent lentiviruses [62–68]. Even though the level of stress may lower in the course of the expedition, it remains high compared to the control.

We suppose that stress induces apoptosis in some cells of the body, including blood cells, as indicated by the high BAX/BCL2 RNA ratio in leukocytes detected in the current study, with the maximal value during the polar night (Figure 7(c)). The upregulation of apoptosis is also confirmed by the elevated cfDNA concentrations (Figure 1(b)), which become high as early as 27 days upon arrival at Vostok; however, then they changed individually. At the same time, we did not note a significant correlation between the BAX/BCL2 RNA ratio and the C_cfDNA_, which has two possible explanations. First, the blood cfDNA pool can be replenished with the DNA fragments from other body cells that died in response to stress [69]. Second, in response to high blood concentration of extracellular DNA, a protective mechanism for its elimination from the circulation is activated. The latter is essential, for a large amount of high-molecular-weight cfDNA negatively affects blood rheology [70]. In addition, cfDNA shows pronounced biological activity toward many cell types. The malfunctioning of the cfDNA elimination system is associated with severe immune pathologies, such as systemic lupus erythematosus [71, 72].

Extracellular DNA is destroyed primarily by blood endonucleases. In this study, the blood NA remained relatively elevated during the whole wintering period, reaching its maximum during the polar night. This fact can be explained by intense apoptosis, which results in a high concentration of cfDNA and claims a more effective cfDNA elimination. The high NA compared to the control, together with the positive correlation between NA and BAX/BCL2 RNA ratio, indicates the persistent entry of additional cfDNA into the bloodstream. In one of our studies, the interconnection between NA and cfDNA concentration (C_cfDNA_) was analyzed in the blood of people exposed to ionizing radiation for a prolonged period of time [25]. We showed elevated NA with decreased C_cfDNA_, which resulted in a low C_cfDNA_/NA ratio compared to the control, while C_cfDNA_ ∗ NA did not differ from the control parameter. For the members of the Antarctic expedition, we noted a significant increase in the latter parameter compared to the control (Figure 3(d)). Apparently, C_cfDNA_ ∗ NA may be used for the assessment of total apoptosis in the body in conditions like Antarctic wintering.

### 4.2. Antarctic Conditions Cause Alterations in cfDNA

The analysis of cfDNA oxidation and the GC/AT ratio serves two purposes. First, these parameters report on the nature and duration of both acute and chronic stress induced by a disease or environmental conditions causing cell death. Second, the content of 8-oxodG and the GC percentage indicate cfDNA biological activity, both positive and pathogenic [47, 73].

In our study, cfDNA oxidation decreased during the wintering (see Figure 4). Similarly, Nirwan et al. reported a decreased 8-oxodG level in the blood of people who practiced yoga in Antarctica [72]. Low oxidation level of cfDNA is associated with its lowered bioactivity and less effective cell penetration [32, 34, 40]. We hypothesize that the elevated NA effectively eliminates the previously oxidized cfDNA fragments from the blood of the expedition members. With that, the pool of extracellular DNA fragments is replenished with the DNA fragments from cells that died for reasons other than DNA oxidation. Because the oxidation of cfDNA decreases with the increase in its concentration, the content of the oxidized fragments remains unchanged and depends both on the oxidation level and the total concentration of cfDNA in the blood.

We assessed the GC percentage by detecting the rDNA repeat in the blood, the content of which increased during the wintering (see Figure 2). Similarly, we observed increased cf-rDNA concentrations in people subjected to ionizing radiation, who had a simultaneous increase in NA and decrease in cfDNA total concentration [44]. Together with the elevated GC percentage, represented by rDNA, the cfDNA of the expedition members is characterized by the decreased concentration of AT-rich fragments, represented by cf-SatIII (Figure 5). The R ratio of the two parameters (cf-rDNA/cf-SatIII) was elevated in all of the subjects during the first half of the wintering (160 days, time points I–III). Interestingly, Antarctic conditions caused the same rise in R as the exposure to ionizing radiation (consider the black curve in Figure 5(c), panel c4). Thus, we propose to use R as a marker of cell death, especially in the conditions of Antarctica. The mechanism underlying the GC/AT disproportion was considered previously [44].

### 4.3. cfDNA Is Potentially an Active Biological Agent

Our in vitro studies on skin fibroblasts demonstrate that plasma cfDNA causes alterations in the expression of genes associated with DNA sensing (*TLR9, AIM2, STING*, and *RIG-I*) [34]. Based on these data, we analyzed the mRNA levels of these genes in the blood leukocytes of the expedition members. The expression of *STING* and *RIG-I* either decreased in some of the subjects or did not change in others, while the changes in the expression of *TLR9* and *AIM2* were individual and multidirectional (Table 3).

*TLR9* gene expression positively correlated with the expression of the proapoptotic gene *BAX* and the concentration of TLR9 ligands in the blood, i.e., the GC-rich fragments of cfDNA (Figures 8 and 9). The significant negative correlation between the mRNA levels of *TLR9* and *STING* in the presence of cfDNA was reported before in *in vitro* studies [36]. This fact may be explained by the cytoplasmic location of STING and RIG-I receptors, while TLR9 receptor can be both in the cell membranes and in endosomes. STING and RIG-I recognize AT-rich regions in the cytoplasmic DNA. During the Antarctic wintering, we observed both low AT content (see Figure 5) and low cfDNA oxidation (Figure 4), which negatively affect cfDNA penetration into the cell. The individual differences in the alterations of cfDNA characteristics detected in the expedition members may be caused by different plasma cfDNA parameters and by alterations in blood cell subpopulations expressing either of the DNA sensors. A high degree of individual variability should be kept in mind while preparing for long polar expeditions or long-term interplanetary missions for implementing a personalized approach in biomedical support. Individual reactions should be studied to increase the efficiency of countermeasures and medicine.

The conditions in Antarctica stimulate the expression of *NFKB1, IL8,* and *IL17A* genes in blood leukocytes, which are the inducers/mediators of inflammation. These data are confirmed by the proinflammatory cytokines in the blood during wintering reported elsewhere [64–70]. The alterations in the expression of these genes positively correlate with the apoptosis markers (see Figure 9), NA level (*NFKB1*, Figure 8(a)), and the R parameter (*IL17A*).

### 4.4. Limitation of the Study

According to the study program, the final blood sample was scheduled at the end of the wintering, a year after the first control sampling. As we demonstrate here and as it is shown by other researchers, adaptive response to the Antarctic conditions develops within the first 100 days (sampling time points I and II). During this very period, all the measured parameters change dramatically, while by the end of the wintering, most of them are similar to the control ones. However, their analysis after the return of the expedition members to normal climate conditions would be quite expedient, as the parameters may change again during the readaptation. Thus, the dynamics of human cfDNA properties in response to the change of a stress-generating environment to a usual habitat may be the topic of another promising research.

## 5. Conclusions

The analyses of cfDNA parameters and the expression of genes associated with cell death and DNA-sensors demonstrated that the Antarctic conditions cause (1) upregulation of apoptosis, which remains active in the first half of the wintering; (2) enrichment of cfDNA with GC fragments, which increase its biological activity; and (3) activation of expression of proinflammatory genes.

## Figures and Tables

**Table 1:** Primer sequences for gene expression analysis.

Gene	F	R
*AIM2*	CAGAAATGATGTCGCAAAGCAA	TCAGTACCATAACTGGCAAACAG
*BAX*	GGAGCTGCAGAGGATGATTG	AGTTGAAGTTGCCGTCAGAA
*BCL2*	TTTGGAAATCCGACCACTAA	AAAGAAATGCAAGTGAATGA
*IL-17A*	TAATGGCCCTGAGGAATGGC	AGGAAGCCTGAGTCTAGGGG
*IL8*	GCACCGACTTTGGAGTTGG	GGACCCCTCAAACGACTGT
*NFKB1*	CAGATGGCCCATACCTTCAAAT	CGGAAACGAAATCCTCTCTGTT
*RIG-I*	GAGATTTTCCGCCTTGGCTAT	CCGTTTCACCTCTGCACTGTT
*STING*	CCAGAGCACACTCTCCGGTA	CGCATTTGGGAGGGAGTAGTA
*TLR9*	CCCACCTGTCACTCAAGTACA	GTGGCTGAAGGTATCGGGATG

**Table 2:** Correlation analysis between the characteristics of blood cfDNA in three groups.

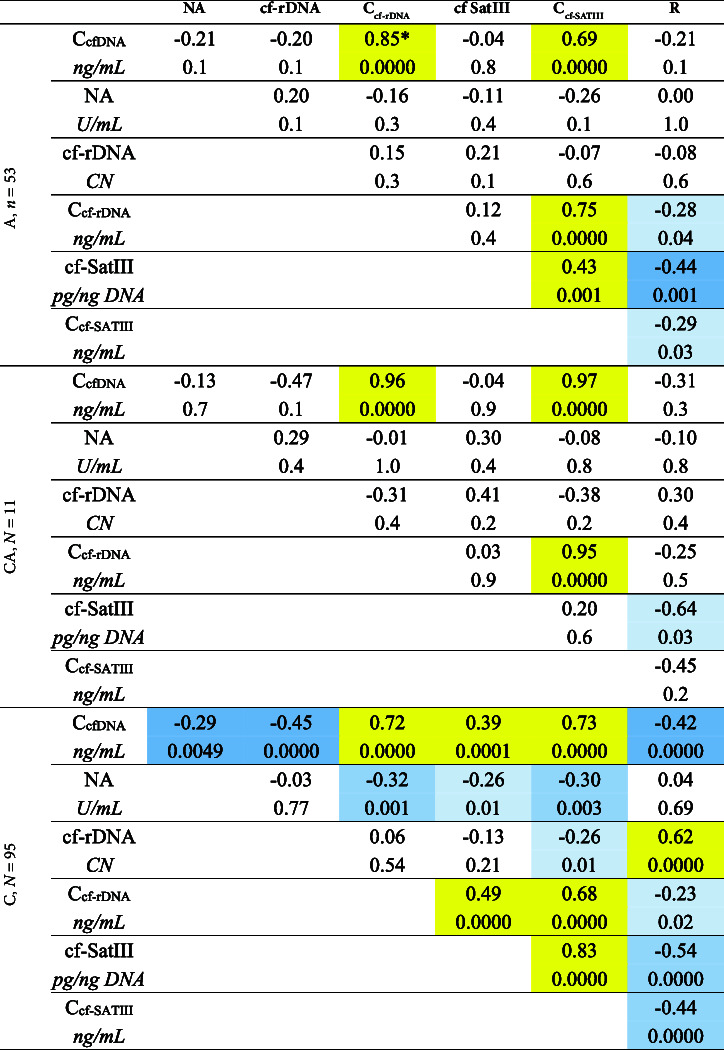

^∗^Yellow cells—positive correlation; blue cells—negative correlation; the probabilities are indicated in each of the cells.

**Table 3:** The analysis of the changes in the expression of DNA-sensor genes (*TLR9, AIM2, STING,* and *RIG-I*) and NF-kB associated genes (*NFKB, IL8,* and *IL17A*).

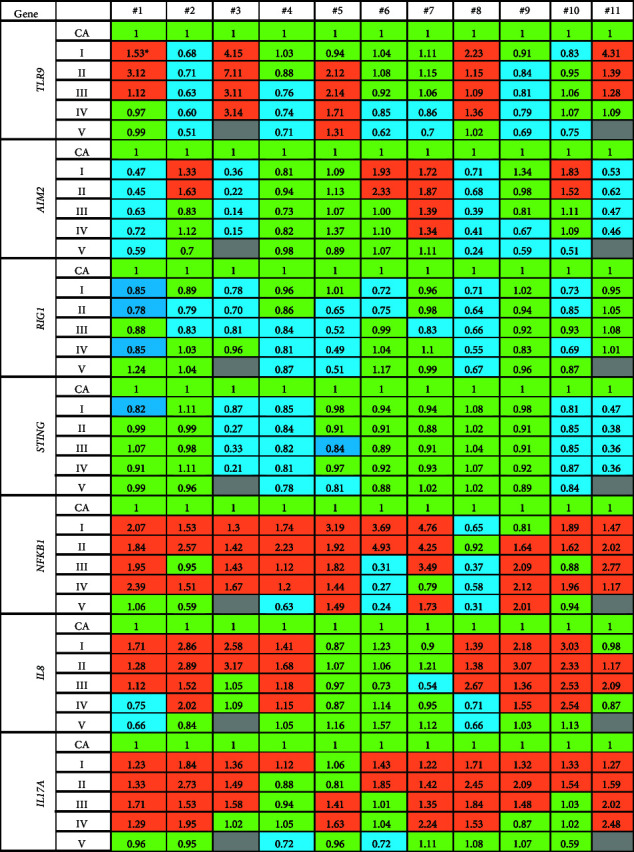

^∗^The mean values of three measurements. The mRNA quantities were normalized to the reference sample (CA). Green cells—the expression did not change compared to control (*p* > 0.05, *U*-test); brown cells—the expression increased (*p* < 0.05); blue cells—the expression decreased (*p* < 0.05).

## Data Availability

The data that support the findings of this study are available in the supporting information of this article.

## Supporting Information

Table S1 demonstrates the correlation analysis between the parameters studied in the work (values normalized to CA control), reflecting the properties of cfDNA and the levels of mRNAs of the genes under study. The analysis was carried out for the entire sample (64 samples) and for each wintering period (I–V).
