# Supplementary material for: The Antarctic Wintering Alters the Properties of Human Plasma Cell-Free DNA
Source: Biochem Res Int. 2025 Sep 9;2025:8994730. doi: 10.1155/bri/8994730 (PMC12440658; doi:10.1155/bri/8994730)
Supplement: Supporting Information — Additional supporting information can be found online in the Supporting Information section. [file 8994730.f1.docx]

Table S1. Correlation analysis between the parameters studied in the work (values normalized to CA control), reflecting the properties of cfDNA and the levels of mRNAs of the genes under study. The analysis was carried out for the entire sample (64 samples) and for each wintering period (I-V).

|  |  | **C cfDNA** | **NA** | **8-oxodG** | **C8-oxodG** | **cf-rDNA** | **C cf-rDNA** | **сf-SatIII** | **Ccf-SatIII** | **R** | ***BAX*** | ***BCL2*** | ***BAX/BCL2*** | ***NFKB*** | ***IL8*** | ***IL17A*** | ***RIG-I*** | ***STING*** | ***TLR9*** | ***AIM2*** |
| --- | --- | --- | --- | --- | --- | --- | --- | --- | --- | --- | --- | --- | --- | --- | --- | --- | --- | --- | --- | --- |
| **C cfDNA** | **n=64** |  | **0.07** | **-0.13** | **0.27** | **-0.11** | **0.93** | **-0.03** | **0.87** | **-0.07** | **-0.11** | **-0.12** | **-0.05** | **0.07** | **0.02** | **0.12** | **0.05** | **-0.36** | **0.24** | **-0.09** |
|  |  |  | **0.6** | **0.3** | **0.031** | **0.4** | **0.0000** | **0.8** | **0.0000** | **0.6** | **0.4** | **0.3** | **0.7** | **0.6** | **0.9** | **0.3** | **0.7** | **0.004** | **0.1** | **0.5** |
| **C cfDNA** | **I** |  | **0.29** | **-0.1** | **0.95** | **-0.32** | **0.98** | **-0.18** | **0.98** | **-0.34** | **-0.37** | **0.04** | **-0.24** | **-0.24** | **-0.25** | **0.03** | **0.16** | **-0.75** | **0.6** | **-0.27** |
|  |  |  | **0.4** | **0.8** | **0.0000** | **0.3** | **0.0000** | **0.6** | **0.0000** | **0.3** | **0.3** | **0.9** | **0.5** | **0.5** | **0.5** | **0.9** | **0.6** | **0.007** | **0.1** | **0.4** |
| **C cfDNA** | **II** |  | **0.5** | **0.2** | **0.59** | **0.38** | **0.72** | **0.38** | **0.7** | **-0.17** | **0.06** | **0.08** | **0.26** | **0.13** | **-0.27** | **0.03** | **0.19** | **0.23** | **-0.28** | **0.19** |
|  |  |  | **0.1** | **0.6** | **0.1** | **0.3** | **0.012** | **0.3** | **0.016** | **0.6** | **0.9** | **0.8** | **0.4** | **0.7** | **0.4** | **0.9** | **0.6** | **0.5** | **0.4** | **0.6** |
| **C cfDNA** | **III** |  | **-0.07** | **-0.1** | **0.57** | **-0.27** | **0.97** | **0.21** | **0.94** | **-0.14** | **-0.47** | **-0.09** | **-0.45** | **0.38** | **0.16** | **0.29** | **0.47** | **-0.56** | **-0.11** | **-0.24** |
|  |  |  | **0.8** | **0.8** | **0.1** | **0.4** | **0.0000** | **0.5** | **0.0000** | **0.7** | **0.1** | **0.8** | **0.2** | **0.3** | **0.6** | **0.4** | **0.1** | **0.1** | **0.7** | **0.5** |
| **C cfDNA** | **IV** |  | **-0.29** | **0.02** | **0.46** | **-0.07** | **0.86** | **0.4** | **0.79** | **-0.21** | **-0.04** | **0.05** | **-0.08** | **0.11** | **0.14** | **0.3** | **-0.17** | **-0.14** | **-0.25** | **0.2** |
|  |  |  | **0.4** | **0.9** | **0.2** | **0.8** | **0.0007** | **0.2** | **0.004** | **0.5** | **0.9** | **0.9** | **0.8** | **0.8** | **0.7** | **0.4** | **0.6** | **0.7** | **0.5** | **0.6** |
| **C cfDNA** | **V** |  | **-0.21** | **0.15** | **0.65** | **-0.04** | **0.82** | **0.51** | **0.85** | **0.07** | **-0.54** | **-0.32** | **-0.3** | **-0.1** | **-0.12** | **-0.07** | **-0.35** | **-0.43** | **0.46** | **0.37** |
|  |  |  | **0.6** | **0.7** | **0.1** | **0.9** | **0.007** | **0.2** | **0.004** | **0.9** | **0.1** | **0.4** | **0.4** | **0.8** | **0.8** | **0.9** | **0.4** | **0.3** | **0.2** | **0.3** |
| **NA** | **n=64** | **0.07** |  | **-0.06** | **-0.04** | **0.19** | **0.13** | **-0.08** | **0.03** | **0.01** | **0.14** | **-0.43** | **0.41** | **0.1** | **0.08** | **0.02** | **-0.09** | **-0.06** | **0.13** | **-0.11** |
|  |  | **0.6** |  | **0.6** | **0.8** | **0.1** | **0.3** | **0.6** | **0.8** | **1.0** | **0.3** | **0.000** | **0.001** | **0.4** | **0.6** | **0.9** | **0.5** | **0.6** | **0.3** | **0.4** |
| **NA** | **I** | **0.29** |  | **0.46** | **0.31** | **0.09** | **0.32** | **0.24** | **0.37** | **-0.33** | **0.1** | **-0.71** | **0.6** | **-0.02** | **0.17** | **-0.19** | **-0.02** | **-0.53** | **-0.04** | **-0.08** |
|  |  | **0.4** |  | **0.2** | **0.4** | **0.8** | **0.3** | **0.5** | **0.3** | **0.3** | **0.8** | **0.014** | **0.1** | **1.0** | **0.6** | **0.6** | **1.0** | **0.1** | **0.9** | **0.8** |
| **NA** | **II** | **0.5** |  | **0.64** | **0.4** | **0.16** | **0.28** | **0.09** | **0.54** | **-0.2** | **-0.26** | **-0.24** | **0.21** | **-0.2** | **-0.35** | **-0.31** | **-0.17** | **0.24** | **0.16** | **-0.31** |
|  |  | **0.1** |  | **0.033** | **0.2** | **0.6** | **0.4** | **0.8** | **0.1** | **0.5** | **0.4** | **0.5** | **0.5** | **0.5** | **0.3** | **0.3** | **0.6** | **0.5** | **0.6** | **0.4** |
| **NA** | **III** | **-0.07** |  | **0.16** | **-0.1** | **0.39** | **0.01** | **0.03** | **-0.06** | **-0.09** | **-0.25** | **-0.21** | **-0.12** | **-0.23** | **0.09** | **-0.26** | **0.39** | **0.19** | **-0.19** | **0.08** |
|  |  | **0.8** |  | **0.6** | **0.8** | **0.2** | **1.0** | **0.9** | **0.9** | **0.8** | **0.5** | **0.5** | **0.7** | **0.5** | **0.8** | **0.4** | **0.2** | **0.6** | **0.6** | **0.8** |
| **NA** | **IV** | **-0.29** |  | **-0.23** | **-0.2** | **-0.4** | **-0.34** | **0.18** | **-0.08** | **-0.19** | **0.12** | **-0.61** | **0.43** | **0.5** | **-0.1** | **-0.27** | **0.03** | **0.02** | **-0.03** | **-0.12** |
|  |  | **0.4** |  | **0.5** | **0.6** | **0.2** | **0.3** | **0.6** | **0.8** | **0.6** | **0.7** | **0.045** | **0.2** | **0.1** | **0.8** | **0.4** | **0.9** | **0.9** | **0.9** | **0.7** |
| **NA** | **V** | **-0.21** |  | **-0.04** | **-0.08** | **0.39** | **0.06** | **-0.05** |  | **-0.2** | **-0.22** | **-0.32** | **0.07** | **-0.26** | **0.3** | **-0.63** | **0.41** | **-0.13** | **0.04** | **-0.01** |
|  |  | **0.6** |  | **0.9** | **0.8** | **0.3** | **0.9** | **0.9** | **0.8** | **0.6** | **0.6** | **0.4** | **0.9** | **0.5** | **0.4** | **0.1** | **0.3** | **0.7** | **0.9** | **1.0** |
| **8-oxodG** | **n=64** | **-0.13** | **-0.06** |  | **0.35** | **0.34** | **-0.02** | **0.3** | **0.07** | **-0.22** | **-0.25** | **0.13** | **-0.19** | **-0.18** | **-0.3** | **-0.29** | **0.06** | **0.22** | **-0.07** | **-0.14** |
|  |  | **0.3** | **0.6** |  | **0.005** | **0.007** | **0.9** | **0.018** | **0.6** | **0.1** | **0.049** | **0.3** | **0.1** | **0.2** | **0.018** | **0.02** | **0.6** | **0.1** | **0.6** | **0.3** |
| **8-oxodG** | **I** | **-0.1** | **0.46** |  | **0.08** | **0.58** | **-0.03** | **0.04** | **-0.04** | **-0.03** | **-0.16** | **-0.57** | **0.43** | **-0.39** | **0.15** | **0.08** | **-0.35** | **-0.02** | **0.21** | **-0.6** |
|  |  | **0.8** | **0.2** |  | **0.8** | **0.1** | **0.9** | **0.9** | **0.9** | **0.9** | **0.6** | **0.1** | **0.2** | **0.2** | **0.7** | **0.8** | **0.3** | **1.0** | **0.5** | **0.05** |
| **8-oxodG** | **II** | **0.2** | **0.64** |  | **0.22** | **0.39** | **0.29** | **0.23** | **0.58** | **-0.4** | **0.07** | **-0.42** | **0.43** | **-0.12** | **-0.14** | **-0.17** | **-0.23** | **0.13** | **0.42** | **-0.37** |
|  |  | **0.6** | **0.033** |  | **0.5** | **0.2** | **0.4** | **0.5** | **0.1** | **0.2** | **0.8** | **0.2** | **0.2** | **0.7** | **0.7** | **0.6** | **0.5** | **0.7** | **0.2** | **0.3** |
| **8-oxodG** | **III** | **-0.1** | **0.16** |  | **0.4** | **0.7** | **0.13** | **-0.23** | **0.03** | **-0.2** | **0.31** | **-0.31** | **0.47** | **0.23** | **-0.27** | **-0.03** | **0.01** | **0.47** | **-0.33** | **0.00** |
|  |  | **0.8** | **0.6** |  | **0.2** | **0.017** | **0.7** | **0.5** | **0.9** | **0.5** | **0.4** | **0.4** | **0.1** | **0.5** | **0.4** | **0.9** | **1.0** | **0.1** | **0.3** | **1.0** |
| **8-oxodG** | **IV** | **0.02** | **-0.23** |  | **0.6** | **0.29** | **0.29** | **0.13** | **0.24** | **-0.49** | **0.16** | **0.13** | **-0.1** | **0.17** | **-0.25** | **-0.34** | **-0.57** | **0.1** | **0.27** | **0.15** |
|  |  | **0.9** | **0.5** |  | **0.1** | **0.4** | **0.4** | **0.7** | **0.5** | **0.1** | **0.6** | **0.7** | **0.8** | **0.6** | **0.5** | **0.3** | **0.1** | **0.8** | **0.4** | **0.7** |
| **8-oxodG** | **V** | **0.15** | **-0.04** |  | **0.26** | **0.78** | **0.5** | **0.02** | **0.27** | **0.2** | **-0.28** | **-0.2** | **-0.17** | **0.44** | **0.03** | **0.32** | **0.19** | **0.27** | **0.19** | **0.43** |
|  |  | **0.7** | **0.9** |  | **0.5** | **0.013** | **0.2** | **1.0** | **0.5** | **0.6** | **0.5** | **0.6** | **0.7** | **0.2** | **0.9** | **0.4** | **0.6** | **0.5** | **0.6** | **0.2** |
| **C*8-oxodG*** | **n=64** | **0.27** | **-0.04** | **0.35** |  | **0.12** | **0.35** | **0.37** | **0.34** | **-0.1** | **-0.15** | **-0.02** | **-0.12** | **0.00** | **-0.16** | **-0.06** | **-0.43** | **-0.01** | **0.09** | **0.08** |
|  |  | **0.031** | **0.8** | **0.005** |  | **0.3** | **0.005** | **0.003** | **0.006** | **0.4** | **0.2** | **0.9** | **0.3** | **1.0** | **0.2** | **0.6** | **0.000** | **0.9** | **0.5** | **0.6** |
| **C*8-oxodG*** | **I** | **0.95** | **0.31** | **0.08** |  | **-0.24** | **0.94** | **-0.35** | **0.95** | **-0.44** | **-0.54** | **0.01** | **-0.3** | **-0.22** | **-0.37** | **-0.05** | **0.19** | **-0.69** | **0.57** | **-0.36** |
|  |  | **0.0000** | **0.4** | **0.8** |  | **0.5** | **0.0000** | **0.3** | **0.0000** | **0.2** | **0.1** | **1.0** | **0.4** | **0.5** | **0.3** | **0.9** | **0.6** | **0.019** | **0.1** | **0.3** |
| **C*8-oxodG*** | **II** | **0.59** | **0.4** | **0.22** |  | **0.06** | **0.25** | **0.18** | **0.37** | **-0.2** | **-0.36** | **0.52** | **-0.32** | **-0.02** | **-0.38** | **-0.47** | **-0.34** | **0.23** | **0.04** | **0.03** |
|  |  | **0.1** | **0.2** | **0.5** |  | **0.9** | **0.5** | **0.6** | **0.3** | **0.6** | **0.3** | **0.1** | **0.3** | **1.0** | **0.3** | **0.1** | **0.3** | **0.5** | **0.9** | **0.9** |
| **C*8-oxodG*** | **III** | **0.57** | **-0.1** | **0.4** |  | **-0.12** | **0.69** | **0.14** | **0.45** | **-0.2** | **-0.37** | **-0.48** | **-0.32** | **0.21** | **-0.23** | **-0.3** | **-0.05** | **-0.08** | **-0.17** | **0.08** |
|  |  | **0.1** | **0.8** | **0.2** |  | **0.7** | **0.019** | **0.7** | **0.2** | **0.5** | **0.3** | **0.1** | **0.3** | **0.5** | **0.5** | **0.4** | **0.9** | **0.8** | **0.6** | **0.8** |
| **C*8-oxodG*** | **IV** | **0.46** | **-0.2** | **0.6** |  | **0.52** | **0.82** | **0.49** | **0.4** | **-0.2** | **0.04** | **0.14** | **-0.09** | **0.05** | **-0.19** | **0.07** | **-0.6** | **0.18** | **0.2** | **0.47** |
|  |  | **0.2** | **0.6** | **0.1** |  | **0.1** | **0.002** | **0.1** | **0.2** | **0.5** | **0.9** | **0.7** | **0.8** | **0.9** | **0.6** | **0.8** | **0.1** | **0.6** | **0.5** | **0.1** |
| **C*8-oxodG*** | **V** | **0.65** | **-0.08** | **0.26** |  | **0.00** | **0.58** | **0.79** | **0.72** | **-0.11** | **-0.21** | **-0.33** | **-0.04** | **0.35** | **0.16** | **0.15** | **-0.63** | **-0.38** | **0.76** | **0.31** |
|  |  | **0.1** | **0.8** | **0.5** |  | **1.0** | **0.1** | **0.011** | **0.03** | **0.8** | **0.6** | **0.4** | **0.9** | **0.4** | **0.7** | **0.7** | **0.1** | **0.3** | **0.017** | **0.4** |
| **cf-rDNA** | **n=64** | **-0.11** | **0.19** | **0.34** | **0.12** |  | **0.22** | **-0.01** | **-0.08** | **0.06** | **0.45** | **-0.17** | **0.45** | **-0.01** | **0.22** | **0.3** | **-0.23** | **0.24** | **0.1** | **-0.11** |
|  |  | **0.4** | **0.1** | **0.007** | **0.3** |  | **0.1** | **1.0** | **0.5** | **0.6** | **0.0002** | **0.2** | **0.0002** | **1.0** | **0.1** | **0.016** | **0.1** | **0.1** | **0.4** | **0.4** |
| **cf-rDNA** | **I** | **-0.32** | **0.09** | **0.58** | **-0.24** |  | **-0.18** | **0.35** | **-0.33** | **0.64** | **0.46** | **-0.27** | **0.57** | **-0.03** | **0.3** | **0.31** | **-0.32** | **0.23** | **0.24** | **-0.41** |
|  |  | **0.3** | **0.8** | **0.1** | **0.5** |  | **0.6** | **0.3** | **0.3** | **0.033** | **0.2** | **0.4** | **0.1** | **0.9** | **0.4** | **0.4** | **0.3** | **0.5** | **0.5** | **0.2** |
| **cf-rDNA** | **II** | **0.38** | **0.16** | **0.39** | **0.06** |  | **0.9** | **0.38** | **0.68** | **-0.15** | **0.63** | **-0.19** | **0.62** | **-0.1** | **0.32** | **0.5** | **-0.34** | **0.21** | **0.06** | **-0.01** |
|  |  | **0.3** | **0.6** | **0.2** | **0.9** |  | **0.0002** | **0.2** | **0.022** | **0.7** | **0.036** | **0.6** | **0.043** | **0.8** | **0.3** | **0.1** | **0.3** | **0.5** | **0.9** | **1.0** |
| **cf-rDNA** | **III** | **-0.27** | **0.39** | **0.7** | **-0.12** |  | **-0.09** | **-0.21** | **-0.16** | **0.00** | **0.55** | **-0.22** | **0.78** | **-0.12** | **0.1** | **0.35** | **-0.09** | **0.55** | **-0.23** | **-0.26** |
|  |  | **0.4** | **0.2** | **0.017** | **0.7** |  | **0.8** | **0.5** | **0.6** | **1.0** | **0.1** | **0.5** | **0.004** | **0.7** | **0.8** | **0.3** | **0.8** | **0.1** | **0.5** | **0.4** |
| **cf-rDNA** | **IV** | **-0.07** | **-0.4** | **0.29** | **0.52** |  | **0.31** | **0.08** | **-0.18** | **0.27** | **0.00** | **0.38** | **-0.2** | **-0.42** | **-0.16** | **-0.09** | **-0.62** | **0.44** | **0.17** | **0.12** |
|  |  | **0.8** | **0.2** | **0.4** | **0.1** |  | **0.3** | **0.8** | **0.6** | **0.4** | **1.0** | **0.2** | **0.6** | **0.2** | **0.6** | **0.8** | **0.042** | **0.2** | **0.6** | **0.7** |
| **cf-rDNA** | **V** | **-0.04** | **0.39** | **0.78** | **0.00** |  | **0.51** | **0.04** | **0.04** | **0.47** | **-0.3** | **-0.16** | **-0.11** | **0.1** | **-0.16** | **0.21** | **0.47** | **0.53** | **0.23** | **0.09** |
|  |  | **0.9** | **0.3** | **0.013** | **1.0** |  | **0.2** | **0.9** | **0.9** | **0.2** | **0.4** | **0.7** | **0.8** | **0.8** | **0.7** | **0.6** | **0.2** | **0.1** | **0.5** | **0.8** |
| **Ccf-rDNA** | **n=64** | **0.93** | **0.13** | **-0.02** | **0.35** | **0.22** |  | **-0.01** | **0.82** | **-0.06** | **0.06** | **-0.21** | **0.14** | **0.09** | **0.1** | **0.23** | **-0.03** | **-0.24** | **0.21** | **-0.04** |
|  |  | **0.0000** | **0.3** | **0.9** | **0.005** | **0.1** |  | **0.9** | **0.0000** | **0.6** | **0.6** | **0.1** | **0.3** | **0.5** | **0.4** | **0.1** | **0.8** | **0.1** | **0.1** | **0.7** |
| **Ccf-rDNA** | **I** | **0.98** | **0.32** | **-0.03** | **0.94** | **-0.18** |  | **-0.15** | **0.95** | **-0.26** | **-0.28** | **0.01** | **-0.15** | **-0.26** | **-0.2** | **0.17** | **0.09** | **-0.68** | **0.58** | **-0.3** |
|  |  | **0.0000** | **0.3** | **0.9** | **0.0000** | **0.6** |  | **0.7** | **0.0000** | **0.4** | **0.4** | **1.0** | **0.7** | **0.4** | **0.5** | **0.6** | **0.8** | **0.021** | **0.1** | **0.4** |
| **Ccf-rDNA** | **II** | **0.72** | **0.28** | **0.29** | **0.25** | **0.9** |  | **0.41** | **0.81** | **-0.14** | **0.53** | **-0.13** | **0.6** | **0.04** | **0.17** | **0.43** | **-0.09** | **0.31** | **-0.18** | **0.17** |
|  |  | **0.012** | **0.4** | **0.4** | **0.5** | **0.0002** |  | **0.2** | **0.003** | **0.7** | **0.1** | **0.7** | **0.05** | **0.9** | **0.6** | **0.2** | **0.8** | **0.4** | **0.6** | **0.6** |
| **Ccf-rDNA** | **III** | **0.97** | **0.01** | **0.13** | **0.69** | **-0.09** |  | **0.18** | **0.9** | **-0.13** | **-0.44** | **-0.25** | **-0.36** | **0.33** | **0.11** | **0.23** | **0.45** | **-0.44** | **-0.19** | **-0.27** |
|  |  | **#####** | **1.0** | **0.7** | **0.019** | **0.8** |  | **0.6** | **0.0001** | **0.7** | **0.2** | **0.5** | **0.3** | **0.3** | **0.8** | **0.5** | **0.2** | **0.2** | **0.6** | **0.4** |
| **Ccf-rDNA** | **IV** | **0.86** | **-0.34** | **0.29** | **0.82** | **0.31** |  | **0.53** | **0.66** | **-0.12** | **0.07** | **0.08** | **-0.03** | **0.06** | **0.09** | **0.18** | **-0.42** | **0.1** | **-0.11** | **0.44** |
|  |  | **0.001** | **0.3** | **0.4** | **0.002** | **0.3** |  | **0.1** | **0.026** | **0.7** | **0.8** | **0.8** | **0.9** | **0.9** | **0.8** | **0.6** | **0.2** | **0.8** | **0.8** | **0.2** |
| **Ccf-rDNA** | **V** | **0.82** | **0.06** | **0.5** | **0.58** | **0.51** |  | **0.55** | **0.74** | **0.31** | **-0.52** | **-0.42** | **-0.18** | **0.03** | **-0.27** | **0.1** | **-0.03** | **-0.06** | **0.58** | **0.27** |
|  |  | **0.007** | **0.9** | **0.2** | **0.1** | **0.2** |  | **0.1** | **0.024** | **0.4** | **0.1** | **0.3** | **0.6** | **0.9** | **0.5** | **0.8** | **0.9** | **0.9** | **0.1** | **0.5** |
| **cf-SATIII** | **n=64** | **-0.03** | **-0.08** | **0.3** | **0.37** | **-0.01** | **-0.01** |  | **0.01** | **0.19** | **-0.18** | **0.29** | **-0.31** | **-0.25** | **-0.29** | **-0.14** | **0.01** | **0.17** | **-0.26** | **-0.11** |
|  |  | **0.8** | **0.6** | **0.018** | **0.003** | **1.0** | **0.9** |  | **0.9** | **0.1** | **0.2** | **0.019** | **0.014** | **0.044** | **0.02** | **0.3** | **0.9** | **0.2** | **0.041** | **0.4** |
| **cf-SATIII** | **I** | **-0.18** | **0.24** | **0.04** | **-0.35** | **0.35** | **-0.15** |  | **-0.22** | **0.41** | **0.75** | **-0.45** | **0.7** | **-0.04** | **0.61** | **0.25** | **-0.08** | **0.08** | **-0.02** | **0.15** |
|  |  | **0.6** | **0.5** | **0.9** | **0.3** | **0.3** | **0.7** |  | **0.5** | **0.2** | **0.007** | **0.2** | **0.017** | **0.9** | **0.044** | **0.5** | **0.8** | **0.8** | **1.0** | **0.7** |
| **cf-SATIII** | **II** | **0.38** | **0.09** | **0.23** | **0.18** | **0.38** | **0.41** |  | **0.36** | **-0.18** | **0.26** | **0.41** | **-0.06** | **-0.17** | **-0.01** | **0.26** | **0.06** | **-0.25** | **0.19** | **-0.43** |
|  |  | **0.3** | **0.8** | **0.5** | **0.6** | **0.2** | **0.2** |  | **0.3** | **0.6** | **0.4** | **0.2** | **0.9** | **0.6** | **1.0** | **0.4** | **0.8** | **0.4** | **0.6** | **0.2** |
| **cf-SATIII** | **III** | **0.21** | **0.03** | **-0.23** | **0.14** | **-0.21** | **0.18** |  | **0.18** | **0.48** | **0.07** | **0.25** | **-0.07** | **0.25** | **-0.19** | **-0.07** | **-0.01** | **0.04** | **-0.18** | **0.49** |
|  |  | **0.5** | **0.9** | **0.5** | **0.7** | **0.5** | **0.6** |  | **0.6** | **0.1** | **0.8** | **0.5** | **0.8** | **0.5** | **0.6** | **0.8** | **1.0** | **0.9** | **0.6** | **0.1** |
| **cf-SATIII** | **IV** | **0.4** | **0.18** | **0.13** | **0.49** | **0.08** | **0.53** |  | **0.28** | **0.13** | **0.07** | **-0.17** | **0.22** | **0.23** | **0.01** | **0.3** | **-0.26** | **0.67** | **-0.65** | **0.7** |
|  |  | **0.2** | **0.6** | **0.7** | **0.1** | **0.8** | **0.1** |  | **0.4** | **0.7** | **0.8** | **0.6** | **0.5** | **0.5** | **1.0** | **0.4** | **0.4** | **0.023** | **0.032** | **0.016** |
| **cf-SATIII** | **V** | **0.51** | **-0.05** | **0.02** | **0.79** | **0.04** | **0.55** |  | **0.4** | **0.18** | **0.01** | **-0.43** | **0.3** | **0.24** | **-0.21** | **0.4** | **-0.37** | **0.00** | **0.67** | **0.00** |
|  |  | **0.2** | **0.9** | **1.0** | **0.011** | **0.9** | **0.1** |  | **0.3** | **0.6** | **1.0** | **0.2** | **0.4** | **0.5** | **0.6** | **0.3** | **0.3** | **1.0** | **0.049** | **1.0** |
| **Ccf-SatIII** | **n=64** | **0.87** | **0.03** | **0.07** | **0.34** | **-0.08** | **0.82** | **0.01** |  | **-0.2** | **-0.21** | **-0.07** | **-0.12** | **-0.03** | **-0.04** | **-0.05** | **0.01** | **-0.25** | **0.24** | **-0.07** |
|  |  | **0.0000** | **0.8** | **0.6** | **0.006** | **0.5** | **0.0000** | **0.9** |  | **0.1** | **0.1** | **0.6** | **0.3** | **0.8** | **0.7** | **0.7** | **0.9** | **0.1** | **0.1** | **0.6** |
| **Ccf-SatIII** | **I** | **0.98** | **0.37** | **-0.04** | **0.95** | **-0.33** | **0.95** | **-0.22** |  | **-0.41** | **-0.43** | **0.01** | **-0.27** | **-0.23** | **-0.22** | **-0.05** | **0.11** | **-0.81** | **0.6** | **-0.26** |
|  |  | **0.0000** | **0.3** | **0.9** | **0.0000** | **0.3** | **######** | **0.5** |  | **0.2** | **0.2** | **1.0** | **0.4** | **0.5** | **0.5** | **0.9** | **0.7** | **0.003** | **0.052** | **0.4** |
| **Ccf-SatIII** | **II** | **0.7** | **0.54** | **0.58** | **0.37** | **0.68** | **0.81** | **0.36** |  | **-0.44** | **0.24** | **-0.23** | **0.52** | **-0.11** | **0.06** | **0.31** | **-0.09** | **0.56** | **-0.18** | **0.00** |
|  |  | **0.016** | **0.1** | **0.1** | **0.3** | **0.022** | **0.003** | **0.3** |  | **0.2** | **0.5** | **0.5** | **0.1** | **0.7** | **0.9** | **0.4** | **0.8** | **0.1** | **0.6** | **1.0** |
| **Ccf-SatIII** | **III** | **0.94** | **-0.06** | **0.03** | **0.45** | **-0.16** | **0.9** | **0.18** |  | **-0.28** | **-0.29** | **0.11** | **-0.31** | **0.59** | **0.11** | **0.4** | **0.55** | **-0.53** | **-0.11** | **-0.19** |
|  |  | **0.0000** | **0.9** | **0.9** | **0.2** | **0.6** | **0.0001** | **0.6** |  | **0.4** | **0.4** | **0.7** | **0.4** | **0.1** | **0.8** | **0.2** | **0.1** | **0.1** | **0.7** | **0.6** |
| **Ccf-SatIII** | **IV** | **0.79** | **-0.08** | **0.24** | **0.4** | **-0.18** | **0.66** | **0.28** |  | **-0.55** | **-0.22** | **-0.11** | **-0.18** | **0.29** | **0.29** | **-0.04** | **-0.43** | **-0.14** | **-0.12** | **0.17** |
|  |  | **0.004** | **0.8** | **0.5** | **0.2** | **0.6** | **0.026** | **0.4** |  | **0.1** | **0.5** | **0.8** | **0.6** | **0.4** | **0.4** | **0.9** | **0.2** | **0.7** | **0.7** | **0.6** |
| **Ccf-SatIII** | **V** | **0.85** | **0.12** | **0.27** | **0.72** | **0.04** | **0.74** | **0.4** |  | **-0.31** | **-0.55** | **-0.53** | **-0.21** | **0.1** | **0.1** | **-0.35** | **-0.36** | **-0.64** | **0.51** | **0.4** |
|  |  | **0.004** | **0.8** | **0.5** | **0.03** | **0.9** | **0.024** | **0.3** |  | **0.4** | **0.1** | **0.1** | **0.6** | **0.8** | **0.8** | **0.4** | **0.3** | **0.1** | **0.2** | **0.3** |
| **R** | **n=64** | **-0.07** | **0.00** | **-0.22** | **-0.1** | **0.06** | **-0.06** | **0.19** | **-0.2** |  | **0.16** | **-0.12** | **0.13** | **0.02** | **0.07** | **0.23** | **-0.06** | **0.12** | **-0.08** | **0.11** |
|  |  | **0.6** | **1.0** | **0.1** | **0.4** | **0.6** | **0.6** | **0.1** | **0.1** |  | **0.2** | **0.3** | **0.3** | **0.9** | **0.6** | **0.1** | **0.6** | **0.3** | **0.5** | **0.4** |
| **R** | **I** | **-0.34** | **-0.33** | **-0.03** | **-0.44** | **0.64** | **-0.26** | **0.41** | **-0.41** |  | **0.63** | **0.17** | **0.32** | **-0.01** | **0.33** | **0.33** | **-0.35** | **0.23** | **0.21** | **-0.12** |
|  |  | **0.3** | **0.3** | **0.9** | **0.2** | **0.033** | **0.4** | **0.2** | **0.2** |  | **0.036** | **0.6** | **0.3** | **1.0** | **0.3** | **0.3** | **0.3** | **0.5** | **0.5** | **0.7** |
| **R** | **II** | **-0.17** | **-0.2** | **-0.4** | **-0.2** | **-0.15** | **-0.14** | **-0.18** | **-0.44** |  | **-0.04** | **-0.12** | **-0.07** | **0.7** | **-0.29** | **0.1** | **-0.14** | **0.00** | **-0.08** | **0.57** |
|  |  | **0.6** | **0.5** | **0.2** | **0.6** | **0.7** | **0.7** | **0.6** | **0.2** |  | **0.9** | **0.7** | **0.8** | **0.016** | **0.4** | **0.8** | **0.7** | **1.0** | **0.8** | **0.1** |
| **R** | **III** | **-0.14** | **-0.09** | **-0.2** | **-0.2** | **0.00** | **-0.13** | **0.48** | **-0.28** |  | **0.12** | **-0.05** | **0.16** | **-0.27** | **0.05** | **0.06** | **-0.05** | **0.24** | **-0.3** | **0.05** |
|  |  | **0.7** | **0.8** | **0.5** | **0.5** | **1.0** | **0.7** | **0.1** | **0.4** |  | **0.7** | **0.9** | **0.6** | **0.4** | **0.9** | **0.9** | **0.9** | **0.5** | **0.4** | **0.9** |
| **R** | **IV** | **-0.21** | **-0.19** | **-0.49** | **-0.2** | **0.27** | **-0.12** | **0.13** | **-0.55** |  | **0.14** | **-0.18** | **0.32** | **-0.35** | **0.26** | **0.33** | **0.32** | **0.49** | **-0.33** | **0.3** |
|  |  | **0.5** | **0.6** | **0.1** | **0.5** | **0.4** | **0.7** | **0.7** | **0.1** |  | **0.7** | **0.6** | **0.3** | **0.3** | **0.4** | **0.3** | **0.3** | **0.1** | **0.3** | **0.4** |
| **R** | **V** | **0.07** | **-0.2** | **0.2** | **-0.11** | **0.47** | **0.31** | **0.18** | **-0.31** |  | **-0.26** | **0.38** | **-0.35** | **-0.37** | **-0.54** | **0.57** | **0.09** | **0.77** | **0.26** | **-0.29** |
|  |  | **0.9** | **0.6** | **0.6** | **0.8** | **0.2** | **0.4** | **0.6** | **0.4** |  | **0.5** | **0.3** | **0.4** | **0.3** | **0.1** | **0.1** | **0.8** | **0.014** | **0.5** | **0.4** |
| ***BAX*** | **n=64** | **-0.11** | **0.14** | **-0.25** | **-0.15** | **0.45** | **0.06** | **-0.18** | **-0.21** | **0.16** |  | **-0.33** | **0.83** | **0.43** | **0.52** | **0.49** | **-0.24** | **-0.14** | **0.28** | **0.1** |
|  |  | **0.4** | **0.3** | **0.049** | **0.2** | **0.0002** | **0.6** | **0.2** | **0.1** | **0.2** |  | **0.007** | **0.0000** | **0.0004** | **0.0000** | **0.0000** | **0.1** | **0.3** | **0.025** | **0.4** |
| ***BAX*** | **I** | **-0.37** | **0.1** | **-0.16** | **-0.54** | **0.46** | **-0.28** | **0.75** | **-0.43** | **0.63** |  | **-0.14** | **0.74** | **0.38** | **0.32** | **0.25** | **-0.12** | **0.22** | **-0.21** | **0.28** |
|  |  | **0.3** | **0.8** | **0.6** | **0.1** | **0.2** | **0.4** | **0.007** | **0.2** | **0.036** |  | **0.7** | **0.009** | **0.3** | **0.3** | **0.5** | **0.7** | **0.5** | **0.5** | **0.4** |
| ***BAX*** | **II** | **0.06** | **-0.26** | **0.07** | **-0.36** | **0.63** | **0.53** | **0.26** | **0.24** | **-0.04** |  | **-0.19** | **0.75** | **0.01** | **0.77** | **0.52** | **0.09** | **-0.33** | **0.33** | **0.00** |
|  |  | **0.9** | **0.4** | **0.8** | **0.3** | **0.036** | **0.1** | **0.4** | **0.5** | **0.9** |  | **0.6** | **0.008** | **1.0** | **0.006** | **0.1** | **0.8** | **0.3** | **0.3** | **1.0** |
| ***BAX*** | **III** | **-0.47** | **-0.25** | **0.31** | **-0.37** | **0.55** | **-0.44** | **0.07** | **-0.29** | **0.12** |  | **0.47** | **0.91** | **0.29** | **-0.12** | **0.52** | **-0.54** | **0.48** | **0.06** | **0.1** |
|  |  | **0.1** | **0.5** | **0.4** | **0.3** | **0.1** | **0.2** | **0.8** | **0.4** | **0.7** |  | **0.1** | **0.0001** | **0.4** | **0.7** | **0.1** | **0.1** | **0.1** | **0.9** | **0.8** |
| ***BAX*** | **IV** | **-0.04** | **0.12** | **0.16** | **0.04** | **0.00** | **0.07** | **0.07** | **-0.22** | **0.14** |  | **-0.46** | **0.86** | **0.62** | **0.26** | **-0.41** | **0.03** | **0.04** | **0.08** | **-0.15** |
|  |  | **0.9** | **0.7** | **0.6** | **0.9** | **1.0** | **0.8** | **0.8** | **0.5** | **0.7** |  | **0.2** | **0.001** | **0.04** | **0.4** | **0.2** | **0.9** | **0.9** | **0.8** | **0.7** |
| ***BAX*** | **V** | **-0.54** | **-0.22** | **-0.28** | **-0.21** | **-0.3** | **-0.52** | **0.01** | **-0.55** | **-0.26** |  | **0.15** | **0.77** | **0.57** | **0.04** | **0.39** | **0.15** | **0.08** | **-0.25** | **-0.19** |
|  |  | **0.1** | **0.6** | **0.5** | **0.6** | **0.4** | **0.1** | **1.0** | **0.1** | **0.5** |  | **0.7** | **0.015** | **0.1** | **0.9** | **0.3** | **0.7** | **0.8** | **0.5** | **0.6** |
| ***BCL2*** | **n=64** | **-0.12** | **-0.43** | **0.13** | **-0.02** | **-0.17** | **-0.21** | **0.29** | **-0.07** | **-0.12** | **-0.33** |  | **-0.66** | **-0.32** | **-0.37** | **-0.29** | **0.1** | **0.09** | **-0.13** | **-0.13** |
|  |  | **0.3** | **0.0004** | **0.3** | **0.9** | **0.2** | **0.1** | **0.019** | **0.6** | **0.3** | **0.007** |  | **0.0000** | **0.011** | **0.003** | **0.021** | **0.4** | **0.5** | **0.3** | **0.3** |
| ***BCL2*** | **I** | **0.04** | **-0.71** | **-0.57** | **0.01** | **-0.27** | **0.01** | **-0.45** | **0.01** | **0.17** | **-0.14** |  | **-0.7** | **0.39** | **-0.47** | **0.08** | **-0.23** | **0.15** | **0.1** | **0.34** |
|  |  | **0.9** | **0.0141** | **0.1** | **1.0** | **0.4** | **1.0** | **0.2** | **1.0** | **0.6** | **0.7** |  | **0.0174** | **0.2** | **0.1** | **0.8** | **0.5** | **0.7** | **0.8** | **0.3** |
| ***BCL2*** | **II** | **0.08** | **-0.24** | **-0.42** | **0.52** | **-0.19** | **-0.13** | **0.41** | **-0.23** | **-0.12** | **-0.19** |  | **-0.71** | **-0.31** | **0.01** | **-0.24** | **-0.14** | **-0.31** | **0.11** | **-0.28** |
|  |  | **0.8** | **0.5** | **0.2** | **0.1** | **0.6** | **0.7** | **0.2** | **0.5** | **0.7** | **0.6** |  | **0.014** | **0.4** | **1.0** | **0.5** | **0.7** | **0.4** | **0.7** | **0.4** |
| ***BCL2*** | **III** | **-0.09** | **-0.21** | **-0.31** | **-0.48** | **-0.22** | **-0.25** | **0.25** | **0.11** | **-0.05** | **0.47** |  | **0.11** | **0.68** | **-0.19** | **0.43** | **-0.05** | **-0.32** | **0.45** | **0.16** |
|  |  | **0.8** | **0.5** | **0.4** | **0.1** | **0.5** | **0.5** | **0.5** | **0.7** | **0.9** | **0.1** |  | **0.8** | **0.022** | **0.6** | **0.2** | **0.9** | **0.3** | **0.2** | **0.6** |
| ***BCL2*** | **IV** | **0.05** | **-0.61** | **0.13** | **0.14** | **0.38** | **0.08** | **-0.17** | **-0.11** | **-0.18** | **-0.46** |  | **-0.81** | **-0.61** | **-0.48** | **0.37** | **-0.04** | **-0.15** | **0.13** | **-0.08** |
|  |  | **0.9** | **0.045** | **0.7** | **0.7** | **0.2** | **0.8** | **0.6** | **0.8** | **0.6** | **0.2** |  | **0.002** | **0.044** | **0.1** | **0.3** | **0.9** | **0.7** | **0.7** | **0.8** |
| ***BCL2*** | **V** | **-0.32** | **-0.32** | **-0.2** | **-0.33** | **-0.16** | **-0.42** | **-0.43** | **-0.53** | **0.38** | **0.15** |  | **-0.48** | **-0.31** | **0.13** | **0.18** | **-0.15** | **0.17** | **-0.06** | **-0.14** |
|  |  | **0.4** | **0.4** | **0.6** | **0.4** | **0.7** | **0.3** | **0.2** | **0.1** | **0.3** | **0.7** |  | **0.2** | **0.4** | **0.7** | **0.7** | **0.7** | **0.7** | **0.9** | **0.7** |
| ***BAX/BCL2*** | **n=64** | **-0.05** | **0.41** | **-0.19** | **-0.12** | **0.45** | **0.14** | **-0.31** | **-0.12** | **0.13** | **0.83** | **-0.66** |  | **0.43** | **0.51** | **0.47** | **-0.2** | **-0.06** | **0.22** | **0.16** |
|  |  | **0.7** | **0.001** | **0.1** | **0.3** | **0.0002** | **0.3** | **0.014** | **0.3** | **0.3** | **0.0000** | **0.0000** |  | **0.0004** | **0.0000** | **0.0001** | **0.1** | **0.7** | **0.1** | **0.2** |
| ***BAX/BCL2*** | **I** | **-0.24** | **0.6** | **0.43** | **-0.3** | **0.57** | **-0.15** | **0.6973** | **-0.27** | **0.32** | **0.74** | **-0.7** |  | **0.05** | **0.37** | **0.13** | **-0.07** | **0.03** | **-0.17** | **-0.11** |
|  |  | **0.5** | **0.05** | **0.2** | **0.4** | **0.1** | **0.7** | **0.0171** | **0.4** | **0.3** | **0.009** | **0.017** |  | **0.9** | **0.3** | **0.7** | **0.8** | **0.9** | **0.6** | **0.7** |
| ***BAX/BCL2*** | **II** | **0.26** | **0.21** | **0.43** | **-0.32** | **0.62** | **0.6** | **-0.06** | **0.52** | **-0.07** | **0.75** | **-0.71** |  | **0.16** | **0.45** | **0.4** | **0.12** | **0.08** | **0.13** | **0.17** |
|  |  | **0.4** | **0.5** | **0.2** | **0.3** | **0.043** | **0.0** | **0.9** | **0.1** | **0.8** | **0.008** | **0.014** |  | **0.6** | **0.2** | **0.2** | **0.7** | **0.8** | **0.7** | **0.6** |
| ***BAX/BCL2*** | **III** | **-0.45** | **-0.12** | **0.47** | **-0.32** | **0.78** | **-0.36** | **-0.07** | **-0.31** | **0.16** | **0.91** | **0.11** |  | **0.02** | **0.03** | **0.5** | **-0.43** | **0.63** | **-0.15** | **-0.06** |
|  |  | **0.2** | **0.7** | **0.1** | **0.3** | **0.004** | **0.3** | **0.8** | **0.4** | **0.6** | **0.0001** | **0.8** |  | **1.0** | **0.9** | **0.1** | **0.2** | **0.036** | **0.7** | **0.9** |
| ***BAX/BCL2*** | **IV** | **-0.08** | **0.43** | **-0.1** | **-0.09** | **-0.2** | **-0.03** | **0.22** | **-0.18** | **0.32** | **0.86** | **-0.81** |  | **0.69** | **0.39** | **-0.3** | **0.11** | **0.18** | **-0.1** | **-0.01** |
|  |  | **0.8** | **0.2** | **0.8** | **0.8** | **0.6** | **0.9** | **0.5** | **0.6** | **0.3** | **0.0007** | **0.002** |  | **0.02** | **0.2** | **0.4** | **0.7** | **0.6** | **0.8** | **1.0** |
| ***BAX/BCL2*** | **V** | **-0.3** | **0.07** | **-0.17** | **-0.04** | **-0.11** | **-0.18** | **0.3** | **-0.21** | **-0.35** | **0.77** | **-0.48** |  | **0.61** | **-0.17** | **0.26** | **0.27** | **0.06** | **-0.14** | **-0.22** |
|  |  | **0.4** | **0.9** | **0.7** | **0.9** | **0.8** | **0.6** | **0.4** | **0.6** | **0.4** | **0.015** | **0.2** |  | **0.1** | **0.7** | **0.5** | **0.5** | **0.9** | **0.7** | **0.6** |
| ***NFKB*** | **n=64** | **0.07** | **0.1** | **-0.18** | **0.00** | **-0.01** | **0.09** | **-0.25** | **-0.03** | **0.02** | **0.43** | **-0.32** | **0.43** |  | **0.01** | **0.2** | **-0.1** | **-0.11** | **0.01** | **0.52** |
|  |  | **0.6** | **0.4** | **0.2** | **1.0** | **1.0** | **0.5** | **0.044** | **0.8** | **0.9** | **0.0004** | **0.011** | **0.0004** |  | **1.0** | **0.1** | **0.4** | **0.4** | **1.0** | **0.0000** |
| ***NFKB*** | **I** | **-0.24** | **-0.02** | **-0.39** | **-0.22** | **-0.03** | **-0.26** | **-0.04** | **-0.23** | **-0.01** | **0.38** | **0.39** | **0.05** |  | **-0.5** | **-0.39** | **0.14** | **0.05** | **-0.35** | **0.56** |
|  |  | **0.5** | **1.0** | **0.2** | **0.5** | **0.9** | **0.4** | **0.9** | **0.5** | **1.0** | **0.3** | **0.2** | **0.9** |  | **0.1** | **0.2** | **0.7** | **0.9** | **0.3** | **0.1** |
| ***NFKB*** | **II** | **0.13** | **-0.2** | **-0.12** | **-0.02** | **-0.1** | **0.04** | **-0.17** | **-0.11** | **0.7** | **0.01** | **-0.31** | **0.16** |  | **-0.38** | **-0.04** | **0.24** | **0.17** | **-0.29** | **0.82** |
|  |  | **0.7** | **0.5** | **0.7** | **1.0** | **0.8** | **0.9** | **0.6** | **0.7** | **0.016** | **1.0** | **0.4** | **0.6** |  | **0.2** | **0.9** | **0.5** | **0.6** | **0.4** | **0.002** |
| ***NFKB*** | **III** | **0.38** | **-0.23** | **0.23** | **0.21** | **-0.12** | **0.33** | **0.25** | **0.59** | **-0.27** | **0.29** | **0.68** | **0.02** |  | **-0.35** | **0.33** | **0.15** | **-0.27** | **0.1** | **0.23** |
|  |  | **0.3** | **0.5** | **0.5** | **0.5** | **0.7** | **0.3** | **0.5** | **0.1** | **0.4** | **0.4** | **0.022** | **1.0** |  | **0.3** | **0.3** | **0.7** | **0.4** | **0.8** | **0.5** |
| ***NFKB*** | **IV** | **0.11** | **0.5** | **0.17** | **0.05** | **-0.42** | **0.06** | **0.23** | **0.29** | **-0.35** | **0.62** | **-0.61** | **0.69** |  | **0.35** | **-0.31** | **-0.17** | **-0.11** | **0.09** | **-0.13** |
|  |  | **0.8** | **0.1** | **0.6** | **0.9** | **0.2** | **0.9** | **0.5** | **0.4** | **0.3** | **0.04** | **0.044** | **0.02** |  | **0.3** | **0.4** | **0.6** | **0.7** | **0.8** | **0.7** |
| ***NFKB*** | **V** | **-0.1** | **-0.26** | **0.44** | **0.35** | **0.1** | **0.03** | **0.24** | **0.1** | **-0.37** | **0.57** | **-0.31** | **0.61** |  | **0.01** | **0.47** | **-0.12** | **-0.05** | **0.18** | **0.1** |
|  |  | **0.8** | **0.5** | **0.2** | **0.4** | **0.8** | **0.9** | **0.5** | **0.8** | **0.3** | **0.1** | **0.4** | **0.1** |  | **1.0** | **0.2** | **0.8** | **0.9** | **0.6** | **0.8** |
| ***IL8*** | **n=64** | **0.02** | **0.08** | **-0.3** | **-0.16** | **0.22** | **0.1** | **-0.29** | **-0.04** | **0.07** | **0.52** | **-0.37** | **0.51** | **0.01** |  | **0.36** | **-0.16** | **-0.1** | **0.2** | **0.08** |
|  |  | **0.9** | **0.6** | **0.018** | **0.2** | **0.1** | **0.4** | **0.02** | **0.7** | **0.6** | **0.0000** | **0.0025** | **0.0000** | **1.0** |  | **0.004** | **0.2** | **0.4** | **0.1** | **0.5** |
| ***IL8*** | **I** | **-0.25** | **0.17** | **0.15** | **-0.37** | **0.3** | **-0.2** | **0.61** | **-0.22** | **0.33** | **0.32** | **-0.47** | **0.37** | **-0.5** |  | **0.46** | **-0.33** | **0.2** | **-0.1** | **0.1** |
|  |  | **0.5** | **0.6** | **0.7** | **0.3** | **0.4** | **0.5** | **0.044** | **0.5** | **0.3** | **0.3** | **0.1** | **0.3** | **0.1** |  | **0.2** | **0.3** | **0.5** | **0.8** | **0.8** |
| ***IL8*** | **II** | **-0.27** | **-0.35** | **-0.14** | **-0.38** | **0.32** | **0.17** | **-0.01** | **0.06** | **-0.29** | **0.77** | **0.01** | **0.45** | **-0.38** |  | **0.4** | **0.00** | **-0.24** | **0.32** | **-0.21** |
|  |  | **0.4** | **0.3** | **0.7** | **0.3** | **0.3** | **0.6** | **1.0** | **0.9** | **0.4** | **0.006** | **1.0** | **0.2** | **0.2** |  | **0.2** | **1.0** | **0.5** | **0.3** | **0.5** |
| ***IL8*** | **III** | **0.16** | **0.09** | **-0.27** | **-0.23** | **0.1** | **0.11** | **-0.19** | **0.11** | **0.05** | **-0.12** | **-0.19** | **0.03** | **-0.35** |  | **0.34** | **0.07** | **-0.01** | **-0.19** | **-0.38** |
|  |  | **0.6** | **0.8** | **0.4** | **0.5** | **0.8** | **0.8** | **0.6** | **0.8** | **0.9** | **0.7** | **0.6** | **0.9** | **0.3** |  | **0.3** | **0.8** | **1.0** | **0.6** | **0.2** |
| ***IL8*** | **IV** | **0.14** | **-0.1** | **-0.25** | **-0.19** | **-0.16** | **0.09** | **0.01** | **0.29** | **0.26** | **0.26** | **-0.48** | **0.39** | **0.35** |  | **-0.26** | **0.05** | **0.2** | **-0.24** | **0.25** |
|  |  | **0.7** | **0.8** | **0.5** | **0.6** | **0.6** | **0.8** | **1.0** | **0.4** | **0.4** | **0.4** | **0.1** | **0.2** | **0.3** |  | **0.4** | **0.9** | **0.6** | **0.5** | **0.5** |
| ***IL8*** | **V** | **-0.12** | **0.3** | **0.03** | **0.16** | **-0.16** | **-0.27** | **-0.21** | **0.1** | **-0.54** | **0.04** | **0.13** | **-0.17** | **0.01** |  | **-0.48** | **0.06** | **-0.56** | **-0.25** | **0.72** |
|  |  | **0.8** | **0.4** | **0.9** | **0.7** | **0.7** | **0.5** | **0.6** | **0.8** | **0.1** | **0.9** | **0.7** | **0.7** | **1.0** |  | **0.2** | **0.9** | **0.1** | **0.5** | **0.028** |
| ***IL17*** | **n=64** | **0.12** | **0.02** | **-0.29** | **-0.06** | **0.3** | **0.23** | **-0.14** | **-0.05** | **0.23** | **0.49** | **-0.29** | **0.47** | **0.2** | **0.36** |  | **-0.18** | **-0.1** | **0.06** | **0.11** |
|  |  | **0.3** | **0.9** | **0.02** | **0.6** | **0.016** | **0.1** | **0.3** | **0.7** | **0.1** | **0.0000** | **0.021** | **0.0001** | **0.1** | **0.004** |  | **0.2** | **0.4** | **0.6** | **0.4** |
| ***IL17*** | **I** | **0.03** | **-0.19** | **0.08** | **-0.05** | **0.31** | **0.17** | **0.25** | **-0.05** | **0.33** | **0.25** | **0.08** | **0.13** | **-0.39** | **0.46** |  | **-0.5** | **0.47** | **0.01** | **0.07** |
|  |  | **0.9** | **0.6** | **0.8** | **0.9** | **0.4** | **0.6** | **0.5** | **0.9** | **0.3** | **0.5** | **0.8** | **0.7** | **0.2** | **0.2** |  | **0.1** | **0.1** | **1.0** | **0.8** |
| ***IL17*** | **II** | **0.03** | **-0.31** | **-0.17** | **-0.47** | **0.5** | **0.43** | **0.26** | **0.31** | **0.1** | **0.52** | **-0.24** | **0.4** | **-0.04** | **0.4** |  | **-0.06** | **0.23** | **-0.25** | **0.18** |
|  |  | **0.9** | **0.3** | **0.6** | **0.1** | **0.1** | **0.2** | **0.4** | **0.4** | **0.8** | **0.1** | **0.5** | **0.2** | **0.9** | **0.2** |  | **0.9** | **0.5** | **0.5** | **0.6** |
| ***IL17*** | **III** | **0.29** | **-0.26** | **-0.03** | **-0.3** | **0.35** | **0.23** | **-0.07** | **0.4** | **0.06** | **0.52** | **0.43** | **0.5** | **0.33** | **0.34** |  | **-0.02** | **-0.25** | **0.22** | **-0.58** |
|  |  | **0.4** | **0.4** | **0.9** | **0.4** | **0.3** | **0.5** | **0.8** | **0.2** | **0.9** | **0.1** | **0.2** | **0.1** | **0.3** | **0.3** |  | **0.9** | **0.5** | **0.5** | **0.1** |
| ***IL17*** | **IV** | **0.3** | **-0.27** | **-0.34** | **0.07** | **-0.09** | **0.18** | **0.3** | **-0.04** | **0.33** | **-0.41** | **0.37** | **-0.3** | **-0.31** | **-0.26** |  | **0.3** | **-0.03** | **-0.18** | **0.23** |
|  |  | **0.4** | **0.4** | **0.3** | **0.8** | **0.8** | **0.6** | **0.4** | **0.9** | **0.3** | **0.2** | **0.3** | **0.4** | **0.4** | **0.4** |  | **0.4** | **0.9** | **0.6** | **0.5** |
| ***IL17*** | **V** | **-0.07** | **-0.63** | **0.32** | **0.15** | **0.21** | **0.1** | **0.4** | **-0.35** | **0.57** | **0.39** | **0.18** | **0.26** | **0.47** | **-0.48** |  | **-0.13** | **0.67** | **0.25** | **-0.22** |
|  |  | **0.9** | **0.1** | **0.4** | **0.7** | **0.6** | **0.8** | **0.3** | **0.4** | **0.1** | **0.3** | **0.7** | **0.5** | **0.2** | **0.2** |  | **0.7** | **0.05** | **0.5** | **0.6** |
| ***RIG1*** | **n=64** | **0.05** | **-0.09** | **0.06** | **-0.43** | **-0.23** | **-0.03** | **0.01** | **0.01** | **-0.06** | **-0.24** | **0.1** | **-0.2** | **-0.1** | **-0.16** | **-0.18** |  | **-0.05** | **-0.28** | **0.02** |
|  |  | **0.7** | **0.5** | **0.6** | **0.0004** | **0.1** | **0.8** | **0.9** | **0.9** | **0.6** | **0.1** | **0.4** | **0.1** | **0.4** | **0.2** | **0.2** |  | **0.7** | **0.023** | **0.9** |
| ***RIG1*** | **I** | **0.16** | **-0.02** | **-0.35** | **0.19** | **-0.32** | **0.09** | **-0.08** | **0.11** | **-0.35** | **-0.12** | **-0.23** | **-0.07** | **0.14** | **-0.33** | **-0.5** |  | **-0.14** | **-0.16** | **-0.08** |
|  |  | **0.6** | **1.0** | **0.3** | **0.6** | **0.3** | **0.8** | **0.8** | **0.7** | **0.3** | **0.7** | **0.5** | **0.8** | **0.7** | **0.3** | **0.1** |  | **0.7** | **0.6** | **0.8** |
| ***RIG1*** | **II** | **0.19** | **-0.17** | **-0.23** | **-0.34** | **-0.34** | **-0.09** | **0.06** | **-0.09** | **-0.14** | **0.09** | **-0.14** | **0.12** | **0.24** | **0.00** | **-0.06** |  | **-0.25** | **-0.37** | **0.12** |
|  |  | **0.6** | **0.6** | **0.5** | **0.3** | **0.3** | **0.8** | **0.8** | **0.8** | **0.7** | **0.8** | **0.7** | **0.7** | **0.5** | **1.0** | **0.9** |  | **0.5** | **0.3** | **0.7** |
| ***RIG1*** | **III** | **0.47** | **0.39** | **0.01** | **-0.05** | **-0.09** | **0.45** | **-0.01** | **0.55** | **-0.05** | **-0.54** | **-0.05** | **-0.43** | **0.15** | **0.07** | **-0.02** |  | **-0.3** | **-0.37** | **-0.07** |
|  |  | **0.1** | **0.2** | **1.0** | **0.9** | **0.8** | **0.2** | **1.0** | **0.1** | **0.9** | **0.1** | **0.9** | **0.2** | **0.7** | **0.8** | **0.9** |  | **0.4** | **0.3** | **0.8** |
| ***RIG1*** | **IV** | **-0.17** | **0.03** | **-0.57** | **-0.6** | **-0.62** | **-0.42** | **-0.26** | **-0.43** | **0.32** | **0.03** | **-0.04** | **0.11** | **-0.17** | **0.05** | **0.3** |  | **-0.32** | **-0.17** | **-0.02** |
|  |  | **0.6** | **0.9** | **0.1** | **0.051** | **0.042** | **0.2** | **0.4** | **0.2** | **0.3** | **0.9** | **0.9** | **0.7** | **0.6** | **0.9** | **0.4** |  | **0.3** | **0.6** | **1.0** |
| ***RIG1*** | **V** | **-0.35** | **0.41** | **0.19** | **-0.63** | **0.47** | **-0.03** | **-0.37** | **-0.36** | **0.09** | **0.15** | **-0.15** | **0.27** | **-0.12** | **0.06** | **-0.13** |  | **0.33** | **-0.63** | **0.21** |
|  |  | **0.4** | **0.3** | **0.6** | **0.1** | **0.2** | **0.9** | **0.3** | **0.3** | **0.8** | **0.7** | **0.7** | **0.5** | **0.8** | **0.9** | **0.7** |  | **0.4** | **0.1** | **0.6** |
| ***STING*** | **n=64** | **-0.36** | **-0.06** | **0.22** | **-0.01** | **0.24** | **-0.24** | **0.17** | **-0.25** | **0.12** | **-0.14** | **0.09** | **-0.06** | **-0.11** | **-0.1** | **-0.1** | **-0.05** |  | **-0.55** | **0.38** |
|  |  | **0.004** | **0.6** | **0.1** | **0.9** | **0.1** | **0.1** | **0.2** | **0.1** | **0.3** | **0.3** | **0.5** | **0.7** | **0.4** | **0.4** | **0.4** | **0.7** |  | **0.0000** | **0.002** |
| ***STING*** | **I** | **-0.75** | **-0.53** | **-0.02** | **-0.69** | **0.23** | **-0.68** | **0.08** | **-0.81** | **0.23** | **0.22** | **0.15** | **0.03** | **0.05** | **0.2** | **0.47** | **-0.14** |  | **-0.6** | **0.33** |
|  |  | **0.007** | **0.1** | **1.0** | **0.019** | **0.5** | **0.021** | **0.8** | **0.003** | **0.5** | **0.5** | **0.7** | **0.9** | **0.9** | **0.5** | **0.1** | **0.7** |  | **0.05** | **0.3** |
| ***STING*** | **II** | **0.23** | **0.24** | **0.13** | **0.23** | **0.21** | **0.31** | **-0.25** | **0.56** | **0.00** | **-0.33** | **-0.31** | **0.08** | **0.17** | **-0.24** | **0.23** | **-0.25** |  | **-0.65** | **0.46** |
|  |  | **0.5** | **0.5** | **0.7** | **0.5** | **0.5** | **0.4** | **0.4** | **0.1** | **1.0** | **0.3** | **0.4** | **0.8** | **0.6** | **0.5** | **0.5** | **0.5** |  | **0.032** | **0.2** |
| ***STING*** | **III** | **-0.56** | **0.19** | **0.47** | **-0.08** | **0.55** | **-0.44** | **0.04** | **-0.53** | **0.24** | **0.48** | **-0.32** | **0.63** | **-0.27** | **-0.01** | **-0.25** | **-0.3** |  | **-0.64** | **0.5** |
|  |  | **0.1** | **0.6** | **0.1** | **0.8** | **0.1** | **0.2** | **0.9** | **0.1** | **0.5** | **0.1** | **0.3** | **0.036** | **0.4** | **1.0** | **0.5** | **0.4** |  | **0.032** | **0.1** |
| ***STING*** | **IV** | **-0.14** | **0.02** | **0.1** | **0.18** | **0.44** | **0.1** | **0.67** | **-0.14** | **0.49** | **0.04** | **-0.15** | **0.18** | **-0.11** | **0.2** | **-0.03** | **-0.32** |  | **-0.66** | **0.64** |
|  |  | **0.7** | **0.9** | **0.8** | **0.6** | **0.2** | **0.8** | **0.023** | **0.7** | **0.1** | **0.9** | **0.7** | **0.6** | **0.7** | **0.6** | **0.9** | **0.3** |  | **0.029** | **0.034** |
| ***STING*** | **V** | **-0.43** | **-0.13** | **0.27** | **-0.38** | **0.53** | **-0.06** | **0.00** | **-0.64** | **0.77** | **0.08** | **0.17** | **0.06** | **-0.05** | **-0.56** | **0.67** | **0.33** |  | **-0.06** | **-0.42** |
|  |  | **0.3** | **0.7** | **0.5** | **0.3** | **0.1** | **0.9** | **1.0** | **0.1** | **0.014** | **0.8** | **0.7** | **0.9** | **0.9** | **0.1** | **0.05** | **0.4** |  | **0.9** | **0.3** |
| ***TLR9*** | **n=64** | **0.24** | **0.13** | **-0.07** | **0.09** | **0.1** | **0.21** | **-0.26** | **0.24** | **-0.08** | **0.28** | **-0.13** | **0.22** | **0.01** | **0.2** | **0.06** | **-0.28** | **-0.55** |  | **-0.41** |
|  |  | **0.1** | **0.3** | **0.6** | **0.5** | **0.4** | **0.1** | **0.041** | **0.1** | **0.5** | **0.025** | **0.3** | **0.1** | **1.0** | **0.1** | **0.6** | **0.023** | **0.0000** |  | **0.0008** |
| ***TLR9*** | **I** | **0.6** | **-0.04** | **0.21** | **0.57** | **0.24** | **0.58** | **-0.02** | **0.6** | **0.21** | **-0.21** | **0.1** | **-0.17** | **-0.35** | **-0.1** | **0.01** | **-0.16** | -0.602 |  | **-0.69** |
|  |  | **0.1** | **0.9** | **0.5** | **0.1** | **0.5** | **0.1** | **1.0** | **0.052** | **0.5** | **0.5** | **0.8** | **0.6** | **0.3** | **0.8** | **1.0** | **0.6** | 0.05 |  | **0.019** |
| ***TLR9*** | **II** | **-0.28** | **0.16** | **0.42** | **0.04** | **0.06** | **-0.18** | **0.19** | **-0.18** | **-0.08** | **0.33** | **0.11** | **0.13** | **-0.29** | **0.32** | **-0.25** | **-0.37** | **-0.65** |  | **-0.59** |
|  |  | **0.4** | **0.6** | **0.2** | **0.9** | **0.9** | **0.6** | **0.6** | **0.6** | **0.8** | **0.3** | **0.7** | **0.7** | **0.4** | **0.3** | **0.5** | **0.3** | **0.032** |  | **0.1** |
| ***TLR9*** | **III** | **-0.11** | **-0.19** | **-0.33** | **-0.17** | **-0.23** | **-0.19** | **-0.18** | **-0.11** | **-0.3** | **0.06** | **0.45** | **-0.15** | **0.1** | **-0.19** | **0.22** | **-0.37** | **-0.64** |  | **-0.44** |
|  |  | **0.7** | **0.6** | **0.3** | **0.6** | **0.5** | **0.6** | **0.6** | **0.7** | **0.4** | **0.9** | **0.2** | **0.7** | **0.8** | **0.6** | **0.5** | **0.3** | **0.032** |  | **0.2** |
| ***TLR9*** | **IV** | **-0.25** | **-0.03** | **0.27** | **0.2** | **0.17** | **-0.11** | **-0.65** | **-0.12** | **-0.33** | **0.08** | **0.13** | **-0.1** | **0.09** | **-0.24** | **-0.18** | **-0.17** | **-0.66** |  | **-0.5** |
|  |  | **0.5** | **0.9** | **0.4** | **0.5** | **0.6** | **0.8** | **0.032** | **0.7** | **0.3** | **0.8** | **0.7** | **0.8** | **0.8** | **0.5** | **0.6** | **0.6** | **0.029** |  | **0.1** |
| ***TLR9*** | **V** | **0.46** | **0.04** | **0.19** | **0.76** | **0.23** | **0.58** | **0.67** | **0.51** | **0.26** | **-0.25** | **-0.06** | **-0.14** | **0.18** | **-0.25** | **0.25** | **-0.63** | **-0.06** |  | **-0.24** |
|  |  | **0.2** | **0.9** | **0.6** | **0.017** | **0.5** | **0.1** | **0.049** | **0.2** | **0.5** | **0.5** | **0.9** | **0.7** | **0.6** | **0.5** | **0.5** | **0.1** | **0.9** |  | **0.5** |
| ***AIM2*** | **n=64** | **-0.09** | **-0.11** | **-0.14** | **0.08** | **-0.11** | **-0.04** | **-0.11** | **-0.07** | **0.11** | **0.1** | **-0.13** | **0.16** | **0.52** | **0.08** | **0.11** | **0.02** | **0.38** | **-0.41** |  |
|  |  | **0.5** | **0.4** | **0.3** | **0.6** | **0.4** | **0.7** | **0.4** | **0.6** | **0.4** | **0.4** | **0.3** | **0.2** | **0.0000** | **0.5** | **0.4** | **0.9** | **0.002** | **0.0008** |  |
| ***AIM2*** | **I** | **-0.27** | **-0.08** | **-0.6** | **-0.36** | **-0.41** | **-0.3** | **0.15** | **-0.26** | **-0.12** | **0.28** | **0.34** | **-0.11** | **0.56** | **0.1** | **0.07** | **-0.08** | **0.33** | **-0.6914** |  |
|  |  | **0.4** | **0.8** | **0.05** | **0.3** | **0.2** | **0.4** | **0.7** | **0.4** | **0.7** | **0.4** | **0.3** | **0.7** | **0.1** | **0.8** | **0.8** | **0.8** | **0.3** | **0.0185** |  |
| ***AIM2*** | **II** | **0.19** | **-0.31** | **-0.37** | **0.03** | **-0.01** | **0.17** | **-0.43** | **0.00** | **0.57** | **0.00** | **-0.28** | **0.17** | **0.82** | **-0.21** | **0.18** | **0.12** | **0.46** | **-0.59** |  |
|  |  | **0.6** | **0.4** | **0.3** | **0.9** | **1.0** | **0.6** | **0.2** | **1.0** | **0.1** | **1.0** | **0.4** | **0.6** | **0.002** | **0.5** | **0.6** | **0.7** | **0.2** | **0.1** |  |
| ***AIM2*** | **III** | **-0.24** | **0.08** | **0.00** | **0.08** | **-0.26** | **-0.27** | **0.49** | **-0.19** | **0.05** | **0.1** | **0.16** | **-0.06** | **0.23** | **-0.38** | **-0.58** | **-0.07** | **0.5** | **-0.44** |  |
|  |  | **0.5** | **0.8** | **1.0** | **0.8** | **0.4** | **0.4** | **0.1** | **0.6** | **0.9** | **0.8** | **0.6** | **0.9** | **0.5** | **0.2** | **0.1** | **0.8** | **0.1** | **0.2** |  |
| ***AIM2*** | **IV** | **0.2** | **-0.12** | **0.15** | **0.47** | **0.12** | **0.44** | **0.7** | **0.17** | **0.3** | **-0.15** | **-0.08** | **-0.01** | **-0.13** | **0.25** | **0.23** | **-0.02** | **0.64** | **-0.5** |  |
|  |  | **0.6** | **0.7** | **0.7** | **0.1** | **0.7** | **0.2** | **0.0** | **0.6** | **0.4** | **0.7** | **0.8** | **1.0** | **0.7** | **0.5** | **0.5** | **1.0** | **0.034** | **0.1** |  |
| ***AIM2*** | **V** | **0.37** | **-0.01** | **0.43** | **0.31** | **0.09** | **0.27** | **0.00** | **0.4** | **-0.29** | **-0.19** | **-0.14** | **-0.22** | **0.1** | **0.72** | **-0.22** | **0.21** | **-0.42** | **-0.24** |  |
|  |  | **0.3** | **1.0** | **0.2** | **0.4** | **0.8** | **0.5** | **1.0** | **0.3** | **0.4** | **0.6** | **0.7** | **0.6** | **0.8** | **0.028** | **0.6** | **0.6** | **0.3** | **0.5** |  |
